# Expression of alternative transcription factor 4 mRNAs and protein isoforms in the developing and adult rodent and human tissues

**DOI:** 10.3389/fnmol.2022.1033224

**Published:** 2022-11-02

**Authors:** Alex Sirp, Anastassia Shubina, Jürgen Tuvikene, Laura Tamberg, Carl Sander Kiir, Laura Kranich, Tõnis Timmusk

**Affiliations:** ^1^Department of Chemistry and Biotechnology, Tallinn University of Technology, Tallinn, Estonia; ^2^Protobios LLC, Tallinn, Estonia

**Keywords:** transcription factor TCF4, basic helix–loop–helix transcription factor, western blot analysis, neurodevelopment, long-read RNA sequencing, brain tissue, peripheral tissue

## Abstract

Transcription factor 4 (TCF4) belongs to the class I basic helix–loop–helix family of transcription factors (also known as E-proteins) and is vital for the development of the nervous system. Aberrations in the *TCF4* gene are associated with several neurocognitive disorders such as schizophrenia, intellectual disability, post-traumatic stress disorder, depression, and Pitt-Hopkins Syndrome, a rare but severe autism spectrum disorder. Expression of the human *TCF4* gene can produce at least 18 N-terminally distinct protein isoforms, which activate transcription with different activities and thus may vary in their function during development. We used long-read RNA-sequencing and western blot analysis combined with the analysis of publicly available short-read RNA-sequencing data to describe both the mRNA and protein expression of the many distinct TCF4 isoforms in rodent and human neural and nonneural tissues. We show that TCF4 mRNA and protein expression is much higher in the rodent brain compared to nonneural tissues. TCF4 protein expression is highest in the rodent cerebral cortex and hippocampus, where expression peaks around birth, and in the rodent cerebellum, where expression peaks about a week after birth. In human, highest *TCF4* expression levels were seen in the developing brain, although some nonneural tissues displayed comparable expression levels to adult brain. In addition, we show for the first time that out of the many possible TCF4 isoforms, the main TCF4 isoforms expressed in the rodent and human brain and other tissues are TCF4-B, -C, -D, -A, and-I. Taken together, our isoform specific analysis of TCF4 expression in different tissues could be used for the generation of gene therapy applications for patients with TCF4-associated diseases.

## Introduction

Transcription factor 4 (TCF4) is a member of the class I basic helix–loop–helix transcription factor family (also known as E-proteins) and is the main E-protein expressed in the adult mouse brain (Massari and Murre, 2000; Fischer et al., 2014). TCF4 regulates numerous genes involved in neurodevelopment (Forrest et al., 2018) and has been shown to mediate its function by forming either homo-or heterodimers with proneural interaction partners such as achaete-scute homolog 1 (ASCL1; Persson et al., 2000) and neurogenic differentiation factor 2 (NEUROD2; Brzózka et al., 2010) as well as negative regulators known as inhibitor of DNA binding (ID) proteins (Chiaramello et al., 1995; Einarson and Chao, 1995). The expression of TCF4 interaction partners is strictly regulated, allowing TCF4 to possibly exert different functions during the development of the nervous system (Quednow et al., 2011).

Changes in the *TCF4* gene are linked to the development of many severe neurocognitive disorders such as schizophrenia (Stefansson et al., 2009; Ripke et al., 2014; Doostparast Torshizi et al., 2019), intellectual disability (Kharbanda et al., 2016), post-traumatic stress disorder (Gelernter et al., 2019), and depression (Wray et al., 2018). In addition, *de novo* mutations in one of the *TCF4* alleles cause Pitt-Hopkins syndrome (Brockschmidt et al., 2007; Zweier et al., 2007)—an autism spectrum disorder described by severe cognitive impairment, breathing abnormalities, motor delay, and distinctive facial features (Zollino et al., 2019). Interestingly, in addition to deletions and translocations, just a single nucleotide mutation in the basic helix–loop–helix encoding domain can completely impair the normal functionality of the TCF4 protein (Amiel et al., 2007; Zweier et al., 2007; Sepp et al., 2012; Sirp et al., 2021). *Tcf4* heterozygous mutant mice exhibit memory deficits, impaired motor control, and social isolation (Kennedy et al., 2016; Thaxton et al., 2018). Similar results have been noted in *Drosophila melanogaster*, where downregulation of Daughterless, the orthologue of TCF4, impairs memory and learning (Tamberg et al., 2020). Overexpression of *Tcf4* in mouse brain causes impairments in cognition and sensorimotor gating (Brzózka et al., 2010), and increased long term depression at synapses (Badowska et al., 2020). Homozygous *Tcf4* knockout mice have low viability and usually die around birth (Zhuang et al., 1996).

Expression of *Tcf4* was first described during late embryonic and early postnatal development in different mouse brain regions using northern blot analysis and *in situ* hybridization (Soosaar et al., 1994; Pscherer et al., 1996; Ravanpay and Olson, 2008). More recent studies have used quantitative droplet digital PCR and reverse-transcription quantitative PCR to show that in the cerebral cortex *TCF4* mRNA expression peaks around birth and declines rapidly in the following 2 weeks (Li et al., 2019; Phan et al., 2020). Expression of TCF4 protein in the developing and adult mouse brain has been described in detail by Jung et al. (2018) using immunostaining with antibodies specific for longer TCF4 protein isoforms. During embryonic development of the brain, expression levels of long TCF4 isoforms are high in the areas which will develop into the cortex and the hippocampus. More specifically, long TCF4 isoforms are largely expressed in the germinal regions that will give rise to GABAergic and glutamatergic neurons of the cortex (Jung et al., 2018). In the brain of adult mice, TCF4 expression of long TCF4 isoforms is high in the cortex, hippocampus, and cerebellum (Jung et al., 2018). Similar results have been obtained by Kim and colleagues who used TCF4-GFP mice to characterize total TCF4 expression (Kim et al., 2020).

In human, TCF4 is expressed broadly, with the expression of different TCF4 isoforms varying between tissues (de Pontual et al., 2009; Sepp et al., 2011). Further analysis of human RNA sequencing (RNA-seq) data has revealed that the mRNA expression dynamics of *TCF4* in human and mouse appear to be conserved in the cerebral cortex—*TCF4* mRNA is highly expressed during fetal stages of development and reaches the maximum before birth, rapidly declines around birth until entering a relatively stable expression level from the early postnatal period to adulthood (Ma et al., 2018). As *TCF4* remains stably expressed in adult humans and rodents alike (de Pontual et al., 2009; Jung et al., 2018; Ma et al., 2018; Li et al., 2019) its expression is probably important for the normal functioning of the organism (Sarkar et al., 2021).

To date, none of the previous studies of *TCF4* mRNA (Ma et al., 2018; Li et al., 2019; Phan et al., 2020) and protein (Jung et al., 2018; Kim et al., 2020) expression have described expression of the variety of TCF4 isoforms. Expression of the mouse and human *TCF4* gene results in many different transcripts encoding N-terminally distinct protein isoforms which vary in their intracellular localization, transactivation capability (Sepp et al., 2011, 2017; Nurm et al., 2021) and possibly mediate their function depending on dosage (Ravanpay and Olson, 2008). Here, we investigated the complex expression dynamics of different TCF4 mRNAs and protein isoforms in the developing and adult rodent and human tissues. Our results can be used to estimate which TCF4 isoforms and at which proportions should be introduced into different tissues during development to generate gene therapy applications of the TCF4-associated diseases.

## Materials and methods

### Direct TCF4 RNA sequencing

Total RNA was extracted from a mixture of cerebral cortices from three P3 BALB/c mice using the RNeasy lipid tissue mini kit (Qiagen). Genomic DNA was digested on-column using RNase-Free DNase Set (Qiagen). Concentration of the purified RNA was determined with BioSpec-nano spectrophotometer (Shimadzu).

Before RNA-seq library preparation, 20 μg of P3 BALB/c cortical RNA was enriched for *Tcf4* transcripts in wash/binding buffer (0.5 M NaCl, 20 mM Tris–HCl pH 7.5, and 1 mM EDTA) with 8 μM each of three 5′ biotinylated oligonucleotides (Microsynth AG)—two oligonucleotides were complementary to the 3` untranslated region of *Tcf4,* and one was complementary to the bHLH region of *Tcf4* (Supplementary Table S1). As there are no known *Tcf4* transcripts that lack the bHLH region or exon 21, we expect that our *Tcf4* mRNA enrichment strategy is unbiased and enriches all possible TCF4 transcripts (Sepp et al., 2011; Nurm et al., 2021). First the mixture was incubated at 70°C for 2 min and then cooled to room temperature in about 30 min in a heating block. When the heating block reached 60°C, 100 units of RiboLock RNase Inhibitor (Thermo Fisher Scientific) was added to the mixture.

Next, Pierce streptavidin magnetic beads (Thermo Fisher Scientific) were prepared for the binding reaction. For that, 40 μl of magnetic beads was washed in 800 μl wash/binding buffer and then suspended in 30 μl of wash/binding buffer. The magnetic beads were then added to the previously annealed oligonucleotide-RNA mixture for the binding reaction. The bead-oligonucleotide-RNA mixture was incubated at RT for 90 min with occasional agitation by hand. After 90 min, the flow-through sample was collected and after that the beads were washed twice with 100 μl of wash/bind buffer followed by three washes with 100 μl of ice-cold low salt buffer (0.15 M NaCl, 20 mM Tris–HCl pH 7.5, and 1 mM EDTA). For elution, the magnetic beads were incubated at 70°C for 5 min in 25 μl of nuclease free water (Qiagen) twice, with the total volume of eluted RNA being 50 μl. In total, two *Tcf4* RNA enrichments were done—the first *Tcf4* enriched RNA sample was sequenced twice and the second once.

RNA sequencing library was prepared separately for each of the three sequencing experiments according to the Sequence-specific direct RNA sequencing protocol SQK-RNA002 (Oxford Nanopore Technologies). The RNAClean XP beads (Agencourt) were substituted with the Mag-Bind total pure NGS magnetic beads (Omega Bio-tek). Sequencing was done three times with the MinION sequencer, FLO-MIN106 flow-cell (new flow cells were used for each experiment), and SQK-RNA002 kit using MinKNOW software (version 3.6.5; Oxford Nanopore Technologies).

Base-calling of the direct RNA sequencing data was performed using Guppy Basecalling Software (version 4.0.11 + f1071ceb, Oxford Nanopore Technologies) with high-accuracy basecalling algorithm. Failed reads were discarded and the passed reads were mapped to mouse GRCm38.p6 genome (obtained from Gencode) using Minimap2 (version 2.17-r941) with the following settings: -ax splice-uf -k14. The generated sam files were converted to bam format using Samtools (version 1.9), the alignments of all three replicates were combined and only reads mapping to the *Tcf4* gene locus were kept. The resulting merged and filtered bam file was then converted to bed12 file format using bedtools (version 2.28.0) for easier visualization. All reads mapping to the *Tcf4* locus in bed12 format can be found in Data Sheet 1. Raw sequencing reads mapping to the *Tcf4* locus can be found in Data Sheet 2. The final data was visualized in Integrated Genomics Viewer and the transcripts encoding *Tcf4* isoforms were manually quantified. Aberrant transcripts were excluded from the analysis.

### Guide RNA design and cloning

The University of California Santa Cruz Genome Browser Gateway^1^ was used to define the genomic region of mouse exon 3 and exon 10a protein coding regions for guide RNA (gRNA) design. The genomic region for mouse exon 3 and exon 10a was chr18:69,347,299–69,347,369 and chr18:69,593,516–69,593,584, respectively, according to mouse GRCm38/mm10 (Dec. 2011) assembly. In total, three gRNAs were designed for exon 3 and two for exon 10a using Benchling Inc.^2^ CRISPR guide design software.

To insert the gRNA targeting region-containing oligonucleotides effectively into the PX459 (Addgene #62988) expression vector, nucleotides were added to the 5` ends of gRNAs that were complementary to the sticky ends produced after restriction of the PX459 plasmid with the BbsI restriction enzyme (Thermo Scientific). In addition, a guanine nucleotide was added to the 5′ end of each forward oligonucleotide sequence of gRNA as it has been found to increase targeting efficiency.^3^ The designed sequences are included in Supplementary Table S2. The oligonucleotides were ordered from Microsynth AG.

### Cell culture and transfection

Mouse Neuro2a and human SH-SY5Y cells were grown in DMEM (Dulbecco’s modified Eagle’s medium, Thermo Scientific) medium, supplemented with 10% fetal bovine serum (Pan Biotech), 100 U/ml penicillin, and 0.1 mg/ml streptomycin (Thermo Scientific).

For transfection, cells were plated on a 12-well plate (Greiner) in 800 μl medium per well 24–48 h before transfection. At the time of the transfection, the cells were at 50–70% confluency. Neuro2a cells were transfected with 500 ng of the pEGFP plasmid and 500 ng of the PX459 plasmid expressing the respective gRNA, Cas9, and Puromycin resistance gene using Lipofectamine 2000 (Invitrogen). In each experiment, DNA to transfection reagent ratio was 1:2. For preparation of protein lysates, the cells were lysed in in 1x Laemmli buffer [0.062 M Tris–HCl pH 6.8, 2% SDS, 5% 2-mercaptoethanol (Roth), 10% glycerol, and 0.01% bromophenol blue].

### Reverse transcription PCR

Total RNA was extracted from Neuro2a cells using the RNeasy mini kit (Qiagen). Genomic DNA was digested on-column using RNase-Free DNase Set (Qiagen). Concentrations of the purified RNAs were determined with BioSpec-nano spectrophotometer (Shimadzu). cDNA was synthesized from Neuro2a total RNA using Superscript IV Reverse Transcriptase (Invitrogen) according to the manufacturer’s instructions. Primers used for reverse transcription PCR are listed in Supplementary Table S3.

### Animal husbandry

The protocols involving animals were approved by the ethics committee of animal experiments at Ministry of Agriculture of Estonia (Permit Number: 45). All experiments were performed in accordance with the relevant guidelines and regulations. WISTAR rats (RccHan:WI, Envigo) and C57BL/6 and BALB/c mouse strains (Envigo) were used in this study. Animals were maintained in conventional polycarbonate or H-TEMP polysulfone cages (2–4 animals per cage) with *ad libitum* access to clean water and food pellets (ssniff Spezialdiäten) under a 12-h light/dark cycle in humidity and temperature-controlled room (temperature 22 ± 1°C and humidity 50 ± 10%).

To establish timed pregnancy for studying embryonic (E) development, the female mouse estrous cycle was monitored by visual observations of the vaginal opening of each female mouse based on the criteria described by Champlin et al. (1973). Mice in the proestrus or estrous phase of the cycle were selected for mating. Animals were bred in the evening and vaginal post-coitum protein plug was checked in the next morning no more than 12 h later. The morning that a plug was found was designated as E0.5 gestational stage. The day of the animal birth was designated as postnatal (P) 0 stage.

### Tissue isolation and protein extraction

Mice and rats were euthanized by carbon dioxide inhalation and decapitated with a guillotine. Dissection of tissue samples was done in ice-cold 1x phosphate-buffered saline solution. Each sample contained tissues pooled together from three different animals for biological diversity and sufficient protein extraction at early developmental stages. The mouse and rat cerebral cortex, hippocampus, cerebellum, olfactory bulb, hypothalamus, and pons including medulla, midbrain, and thalamus were collected. Striatum was collected only for the BALB/c mouse strain. Tissue collection for mouse and rat brain regions occurred at P0, 3, 5, 7, 10, 14, 21, 60, and P0, 3, 5, 10, 14, 30, 60, respectively. In addition to postnatal days, collection of total mouse brain samples occurred at E13, 15, and 18. Mouse and rat peripheral tissues, skin, lung, kidney, heart, diaphragm, muscle, bladder, stomach, pancreas, thymus, spleen, liver, and blood, were collected at developmental stages P0, 14, and 60. After collection, tissue samples were stored at −80°C until further processing.

Tissues were homogenized on ice with tissue grinder PELLET PESTLE® Cordless Motor (Kimble-Chase, DWK Life Sciences) in ice-cold Radioimmunoprecipitation assay buffer [RIPA, 50 mM Tris pH 8.0, 150 mM NaCl, 1% NP-40, 0.5% Na-deoxycholate, 0.5% sodium dodecyl sulfate (SDS), and 1x Roche Protease Inhibitor Cocktail Complete]. Lysates were sonicated for 15 s with Torbeo Ultrasonic probe sonicator (36810-series, Cole Parmer), and insoluble material was removed by centrifugation at 4°C for 20 min at 16,000 *g*. Protein concentration was measured with Pierce BCA Protein Assay Kit (Thermo Scientific).

Protein lysates from human post-mortem cerebral cortex and hippocampus were prepared like rodent lysates. All protocols using human tissue samples were approved by Tallinn Committee for Medical Studies, National Institute for Health Development (Permit Number 402). All experiments were performed in accordance with relevant guidelines and regulations.

### *In vitro* protein translation

TCF4-A^+^, -A^−^, -B^+^, B^−^, -C^−^, -D^−^ and TCF4-I^−^isoforms were translated *in vitro* using pcDNA3 plasmids encoding the respective TCF4 isoforms (Sepp et al., 2011) with TnT Quick Coupled Transcription/Translation System (Promega). Equal volumes of *in vitro* translated TCF4 protein mixtures were used for western blot analysis.

### Western blot analysis

For western blot analysis, protein lysates in RIPA were diluted to the same concentration in 1x Laemmli buffer. 55 μg of each sample was electrophoretically separated by SDS-polyacrylamide gel electrophoresis in 8% gel and transferred to polyvinylidene difluoride membrane (Millipore) in Towbin buffer (25 mM Tris, 192 mM glycine, 20% methanol, 0.1% SDS, and pH 8.3) using wet transfer. Membranes were blocked with 5% skimmed milk (Sigma-Aldrich) in 1x Tris Buffered Saline with 0.1% Tween-20 (TBST, Sigma Aldrich) before incubating with primary [mouse monoclonal anti-ITF-2 (TCF4); C-8, 1:1,000 dilution, Santa Cruz] and secondary (goat polyclonal anti-mouse IgG HRP-conjugated antibody 1:5,000 dilution, Thermo Scientific) antibody in 2.5% skimmed milk-TBST solution overnight at 4°C and 1 h at room temperature, respectively. Specificity of the anti-ITF-2 (TCF4; C-8, Santa Cruz) antibody has been previously validated using tissue lysates from *Tcf4* knockout mice (Nurm et al., 2021). For peripheral tissues, mouse IgG kappa binding protein conjugated to HRP was used as a secondary antibody (1:5,000 dilution, Santa Cruz). Chemiluminescence signal detection with SuperSignal West Femto or Atto Chemiluminescence Substrate (Thermo Scientific). The chemiluminescence signal was visualized with ImageQuant LAS 4000 bioimager (GE Healthcare) and densitometric quantification was performed with ImageQuant TL 8.2 image analysis software (GE Healthcare). Membrane staining with Coomassie solution (0.1% Coomassie Brilliant Blue R-250, 25% ethanol, and 7% acetic acid) was used as a loading control and for total protein normalization.

### Analysis of publicly available RNA-seq datasets

Raw RNA-seq datasets of human, mouse, and rat were obtained from EMBL-EBI European Nucleotide Archive database using www.sra-explorer.info (Keane et al., 2011; ENCODE Project Consortium, 2012; Schmitt et al., 2014; Yu et al., 2014; Vied et al., 2016; Li et al., 2017; Söllner et al., 2017; Cardoso-Moreira et al., 2019; Luo et al., 2020; Shafik et al., 2021; see Supplementary Table S4 for accession numbers and sample information). Adapter and quality trimming were done using BBDuk (part of BBMap version 38.90) with the following parameters: ktrim = r k = 23 mink = 11 hdist = 1 tbo qtrim = lr trimq = 10 maq = 10 minlen = 25. Mouse reads were mapped to mm10 (primary assembly and annotation obtained from GENCODE, release M25, GRCm38), rat reads were mapped to rn6 (primary assembly and annotation obtained from Ensembl, release 104, RGSC 6.0/Rnor_6.0), and human reads were mapped to hg19 (primary assembly and annotation obtained from GENCODE, release 37, GRCh37) using STAR aligner (version 2.7.4a) with default parameters. To increase sensitivity for unannotated splice junctions, splice junctions obtained from the first pass were combined per dataset and filtered as follows: junctions on mitochondrial DNA and non-canonical intron motifs were removed; only junctions detected in at least 10% of samples (rounded up to the nearest integer) in the whole dataset were kept. Filtered junctions were added to the second pass mapping using STAR. Intron spanning reads were quantified using FeatureCounts (version 2.0.1). The following parameters were used for paired-end data: -p –B –C –J; and single-end data: -J. To count reads from *TCF4* extended exons (exons 4c and 7bII), reads crossing a 1 bp region 2 bp 5′ from the internal exon 4 and 7, respectively, were quantified using FeatureCounts and a custom-made saf file. Splice junctions in the *TCF4* locus were manually curated and annotated to TCF4 isoforms according to Sepp et al. (2011).

A custom R script^4^ was used to quantify the expression of different *TCF4* transcripts from analyzed RNA-seq datasets. Briefly, RNA-seq reads crossing the indicated *TCF4* splice-junctions were normalized using all splice-junction crossing reads in the respective sample. Then, the data were summarized by the Exon column (Supplementary Tables S5–S7). To acquire total *TCF4* mRNA levels, the mean value of exon-junctions from 10–11 to 19–20 was taken for the analysis. Aggregated mouse and rat data was meta-analyzed in tandem with human data to study *TCF4* expression during development. The expression of *TCF4* transcripts encoding specific isoforms was assessed by quantifying the number of reads crossing the exon-junctions specific for the TCF4 isoforms. Data were summarized in each tissue and age group by the Isoform column. Values for each isoform were then divided with the sum of all annotated isoforms to show isoform composition in percentages. The results were visualized using ggplot2 (version 3.3.5) in R (version 4.1.2). The *TCF4* exon-junction data used for the analysis of *TCF4* transcripts in mouse, rat, and human datasets can be found in Supplementary Tables S5–S7, respectively.

Data mining and visualization was also performed on human GTEx portal exon-exon junction dataset (dbGaP Accession phs000424.v8.p2) and human developmental transcriptome data from BrainSpan (RNA-Seq Gencode v10 summarized to genes). The human GTEx data used for the analyses described in this manuscript were obtained from the GTEx Portal^5^ on 12/01/2021 and the human BrainSpan data was obtained from the BrainSpan Atlas of the Developing Human Brain^6^ on 12/01/2021. For information about rodent and human developmental stages and the number of individual data points per developmental stage, see Supplementary Tables S8–S12.

## Results

### Five N-terminally distinct TCF4 protein isoforms are expressed in the developing mouse cerebral cortex

**Figure 1:**
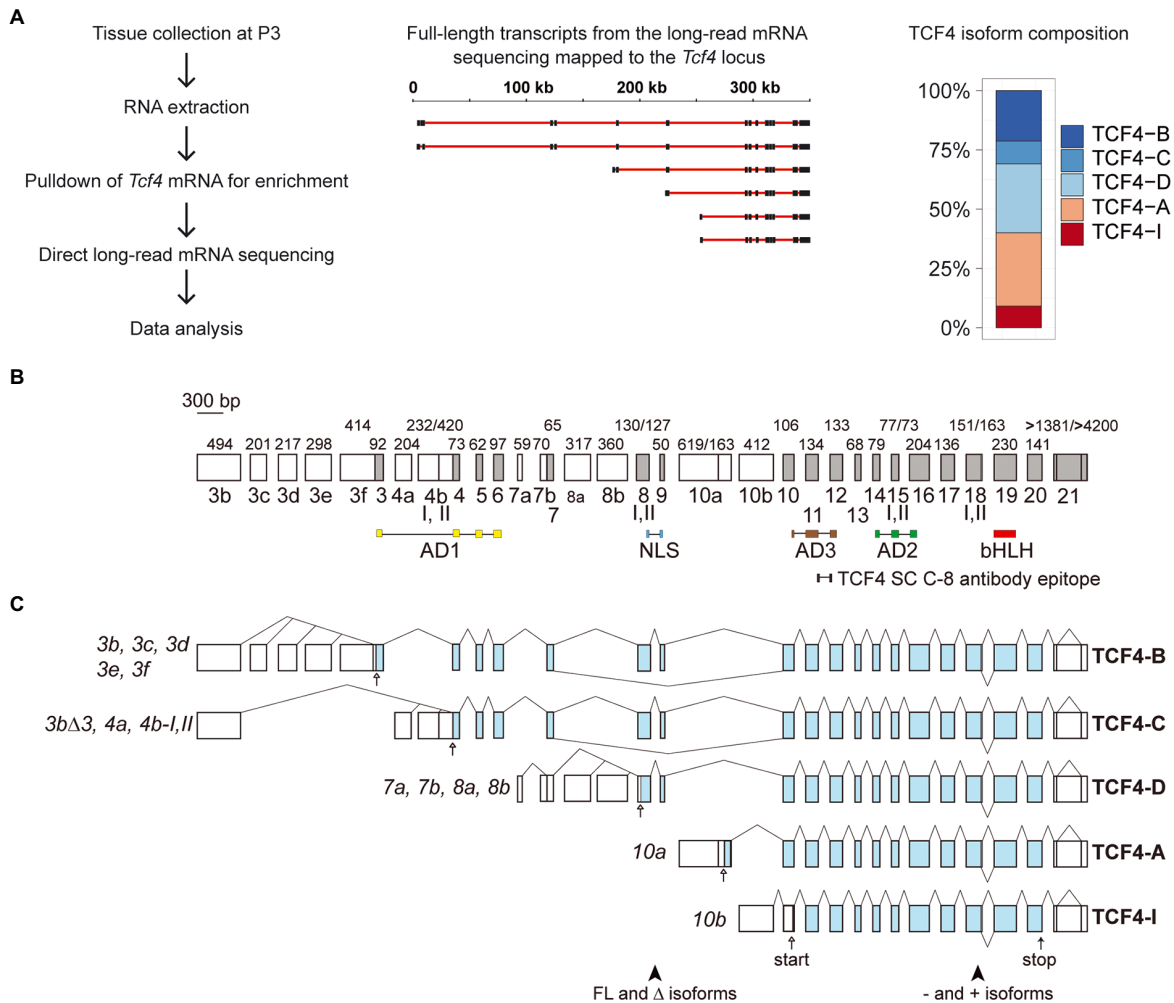
*Tcf4* mRNA isoforms expressed in the developing mouse cerebral cortex encode five N-terminally distinct protein isoforms. **(A)** Schema of experimental design for direct RNA-sequencing. The cerebral cortices of postnatal day 3 (P3) mice were collected, followed by RNA extraction and *Tcf4* mRNA enrichment before direct long-read mRNA sequencing. A selection of *Tcf4* transcripts mapped to the mouse *Tcf4* locus is shown for reference. Black boxes represent exons and red lines show introns. Scale bar in kilobases is shown on top. *Tcf4* transcripts encoding the different TCF4 isoforms (TCF4-B, -C, -D, -A, and-I) were quantified and the distribution is shown on the right. Each isoform is represented with different color as shown in the legend on the right. **(B)** Mouse *Tcf4* genomic organization with exons drawn in scale. Exons are named according to the human *TCF4* gene (Sepp et al., 2011). 5′ exons are shown as white boxes while internal and 3′ exons are shown as gray boxes. Exon names are shown below boxes. Numbers above the exons designate the size of the exon in base pairs. Roman numerals below exons show alternative splice sites. Regions encoding different domains are marked below the gene structure (AD1, NLS, AD3, AD2, and bHLH) as well as the epitope for the TCF4 antibody C-8 (Santa Cruz, SC) used in the present study. **(C)** Schematic structure of *Tcf4* transcripts expressed in the developing mouse cerebral cortex. Untranslated regions are shown as white boxes and translated regions as blue boxes. Each transcript is named (shown on the left) according to the 5′ exon and with the number of the splice site where indicated. The names of the protein isoforms encoded by the transcripts are shown on the right. Positions of alternative splice region that generates full-length (FL), Δ, − and + isoforms are shown at the bottom. The position of the first in-frame start codon is shown with an arrow for each transcript and the common stop codon with an arrow at the bottom. AD, activation domain; NLS, nuclear localization signal; bHLH, basic helix–loop–helix; FL, full-length; and Δ, lack of exons 8–9.

The use of numerous alternative 5′ exons results in the expression of many transcripts from the *Tcf4* gene, resulting in a variety of TCF4 protein isoforms with different expression patterns between tissue types (Sepp et al., 2011; Nurm et al., 2021). We have previously described transcripts from the mouse *Tcf4* gene based on available mRNA and expressed sequence tag data from various tissues (Nurm et al., 2021) but characterizing all the isoform encoding transcripts using short-read sequencing is complicated. Here, we did long-read direct RNA-sequencing (RNA-seq) on the Oxford Nanopore Technologies platform from postnatal day 3 (P3) mouse cerebral cortex. Direct RNA-seq eliminates the bias which may result from complementary DNA synthesis used in conventional RNA-seq methods. Briefly, we extracted total RNA from the P3 mouse cerebral cortex which was then enriched for *Tcf4* transcripts using a combination of oligonucleotides complementary to the 3′ untranslated region and the basic helix–loop–helix region of *Tcf4* (Figure 1A; Supplementary Table S1). The Oxford Nanopore Technologies platform begins sequencing from the 3′ end of RNA meaning that early sequencing termination can result in reads which do not reach the 5′ exons of *Tcf4* transcripts. Analysis of our results showed that most of the 1,336 RNAs which mapped to the mouse *Tcf4* gene were short and mapped only to the last exon of *Tcf4* gene. However, we obtained 163 *Tcf4* transcripts that reached from the 3′untranslated region to the 5′ terminal exons and were thus considered full-length based on previous knowledge about the rodent *Tcf4* gene structure (Nurm et al., 2021). We then quantified the potential TCF4 protein isoforms encoded by these transcripts (Figure 1A). The results showed that in the mouse P3 cerebral cortex ~20% of mRNAs transcribed from *Tcf4* gene encode isoform TCF4-B; 10% encode isoform TCF4-C; 30% encode isoform TCF4-D; 30% encode isoform TCF4-A and 10% encode isoform TCF4-I (Figure 1A). The presence of plus (containing the RSRS amino acid sequence) and minus (without the RSRS amino acid sequence; Corneliussen et al., 1991; Nurm et al., 2021) TCF4 isoforms encoded by *Tcf4* mRNAs was roughly equal. Overall, our data show that the expression of mouse *Tcf4* gene leads to numerous transcripts due to the presence of 32 exons out of which 13 are alternative 5′ exons and 18 are internal exons and one is a terminal 3′ exon (Figures 1B,C). Using long-read sequencing, we discovered two novel 5′ exons (exon 3e and 3f) that are included in *Tcf4* transcripts encoding TCF4-B isoform (Figures 1B,C).

**Figure 2:**
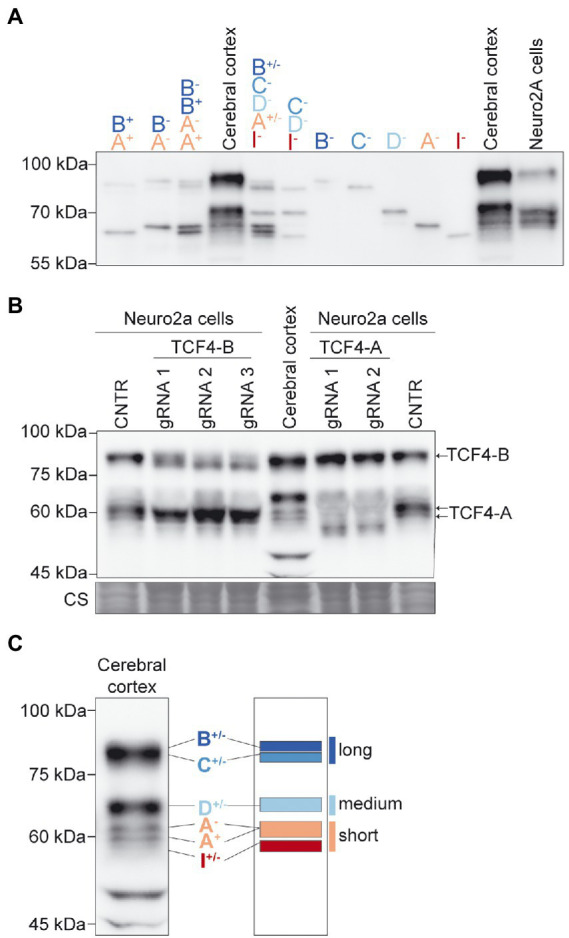
TCF4 protein isoforms expressed in the mouse cerebral cortex can be grouped into long, medium, and short isoforms based on apparent molecular weight in SDS-PAGE. **(A)** Western blot analysis of different combinations of *in vitro* translated TCF4 isoforms (shown on the top) to identify the mobility of TCF4 isoforms in the lysates from the mouse cerebral cortex and Neuro2a cells in SDS-PAGE. **(B)** Western blot analysis of Neuro2a cells transfected with CRISPR-Cas9 silencing system. Two exon 10a-specific gRNAs were used to silence TCF4-A and three exon 3-specific gRNAs were used to silence TCF4-B. Cells overexpressing CRISPR-Cas9 vector without the exon-specific gRNA targeting sequence was used as control (CNTR). Mouse cerebral cortex tissue lysate was used to compare TCF4 isoform expression pattern to the pattern in Neuro2a cells. Locations of TCF4-A and TCF4-B isoforms are depicted on the right. Coomassie staining (CS) was used a loading control and is shown at the bottom. **(C)** Schematic layout of the locations of TCF4 isoforms in the protein lysate of the mouse cerebral cortex in western blot. TCF4 isoforms were grouped into three—long, medium and short isoforms. The locations of TCF4 isoform groups are color coded and shown on the right. In each panel, molecular weight is shown on the left in kilodaltons.

We then described the expression pattern of TCF4 protein isoforms in SDS-PAGE/western blot analysis. For that, we used *in vitro* translated TCF4-B, -C, -D, -A, and-I plus or minus TCF4 isoforms. This allowed us to compare different combinations of *in vitro* translated TCF4 isoforms in western blot to the TCF4 protein pattern in the mouse cerebral cortex. In good agreement with our RNA-seq experiment, the combination of *in vitro* translated proteins TCF4-B, -C, -D, -A, and -I resembled the protein pattern of TCF4 in the cerebral cortex (Figure 2A). The variability in *in vitro* translated TCF4 isoform levels in western blot (Figure 2A) could arise from differential translation rate of the respective TCF4 isoform encoding plasmids. Next, we determined the apparent molecular weight and location of endogenously expressed TCF4 isoforms—TCF4-B and TCF4-A in SDS-PAGE/western blot analysis. To this end, we constructed a CRISPR-Cas9 system to inhibit the expression of these TCF4 isoforms in Neuro2a cell line by generating frameshift mutations in the unique exons encoding these isoforms (exons 3 and 10a, respectively). We could not specifically silence the expression of TCF4-D since its translation start site is in internal exon 8—a frameshift mutation in exon 8 would cause the silencing of not only TCF4-D, but also TCF4-B and-C isoforms. We used Neuro2a cells, which show high endogenous expression of TCF4 as confirmed by RT-PCR and western blot analysis (Figure 2B; Supplementary Figure S1). By expressing the generated CRISPR-Cas9 system in Neuro2a cells, we were able to inhibit the expression of TCF4-B and-A and thus confirm the location of these protein isoforms in western blot analysis (Figure 2B). Furthermore, western blot analysis showed that expression pattern of TCF4 isoforms was similar in Neuro2a cells and P3 mouse cerebral cortex (Figure 2B).

Taken together, the main N-terminally distinct TCF4 isoforms expressed in the early postnatal mouse cerebral cortex are TCF4-B, -C, -D, -A, and -I. We classified the detected TCF4 signals into three groups based on their molecular weight: long isoforms (TCF4-B and-C), medium isoforms (TCF4-D), and short isoforms (TCF4-A and-I; Figure 2C).

### Expression of TCF4 protein in the mouse brain is highest around birth

**Figure 3:**
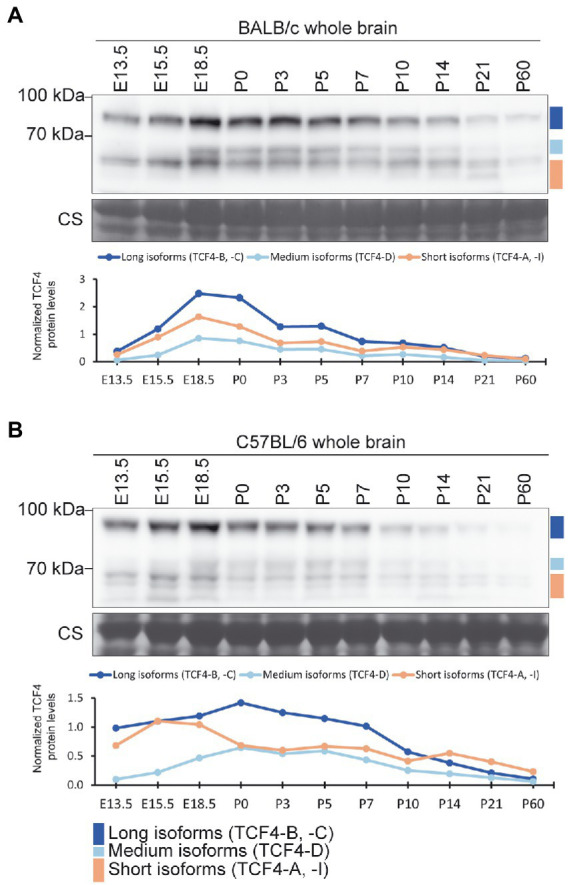
Protein expression of TCF4 in the mouse brain peaks around birth. **(A,B)** Western blot analysis of TCF4 protein expression through the pre-and postnatal development in the BALB/c **(A)** and C57BL/6 **(B)** mouse whole brain. Tissue lysates from the whole brain were made at different embryonic (E) and postnatal (P) days. The locations of TCF4 isoform groups are color coded and shown on the right. In each panel, molecular weight is shown on the left in kilodaltons. Under each western plot panel, a line graph depicting the quantification of long, medium, and short TCF4 protein isoforms during development is shown. TCF4 protein levels were normalized to the total TCF4 signal of the P10 cerebral cortex of the respective mouse strain.

Next, we studied the changes in TCF4 protein expression in the mouse brain throughout pre-and postnatal development. For that, we made mouse whole brain lysates from two strains (BALB/C and C57BL/6) at 11 different developmental stages ranging from E13.5 to P60, and performed western blot analysis (Figures 3A,B). Both mouse strains exhibited expression of long, medium, and short TCF4 isoforms, with the highest expression of total TCF4 detected at late prenatal and early postnatal development. After peaking, TCF4 expression gradually declined during postnatal development of the brain (Figures 3A,B). While long and short TCF4 isoforms were detected at all stages, the medium-sized TCF4 isoforms became more apparent at later fetal stages and were almost undetectable before stage E18.5 (Figures 3A,B).

To better compare TCF4 total levels and isoform expression patterns between the two mouse strains, brain samples of the two strains from early postnatal development (P0–10) were analyzed in the same western blot experiment (Supplementary Figure S2). Our results revealed that the two mouse strains showed no major differences in TCF4 expression levels or expression patterns (Supplementary Figure S2). Altogether, these results indicated that TCF4 is expressed at both pre-and postnatal stages of the mouse brain development, with long and short TCF4 isoforms presented at all stages.

### Expression of TCF4 is highest in the cerebral cortex, hippocampus, cerebellum, and olfactory bulb in the rodent brain

Next, we analyzed mRNA and protein expression of TCF4 in various rodent brain regions. First, we conducted a meta-analysis of available short-read RNA-seq data (Keane et al., 2011; ENCODE Project Consortium, 2012; Schmitt et al., 2014; Yu et al., 2014; Vied et al., 2016; Li et al., 2017; Söllner et al., 2017; Cardoso-Moreira et al., 2019; Luo et al., 2020; Shafik et al., 2021) to quantify the expression of total *Tcf4* mRNA and different *Tcf4* transcripts encoding distinct protein isoforms in rodents. Where possible, *Tcf4* expression dynamics was studied during different stages of pre-and postnatal development.

**Figure 4:**
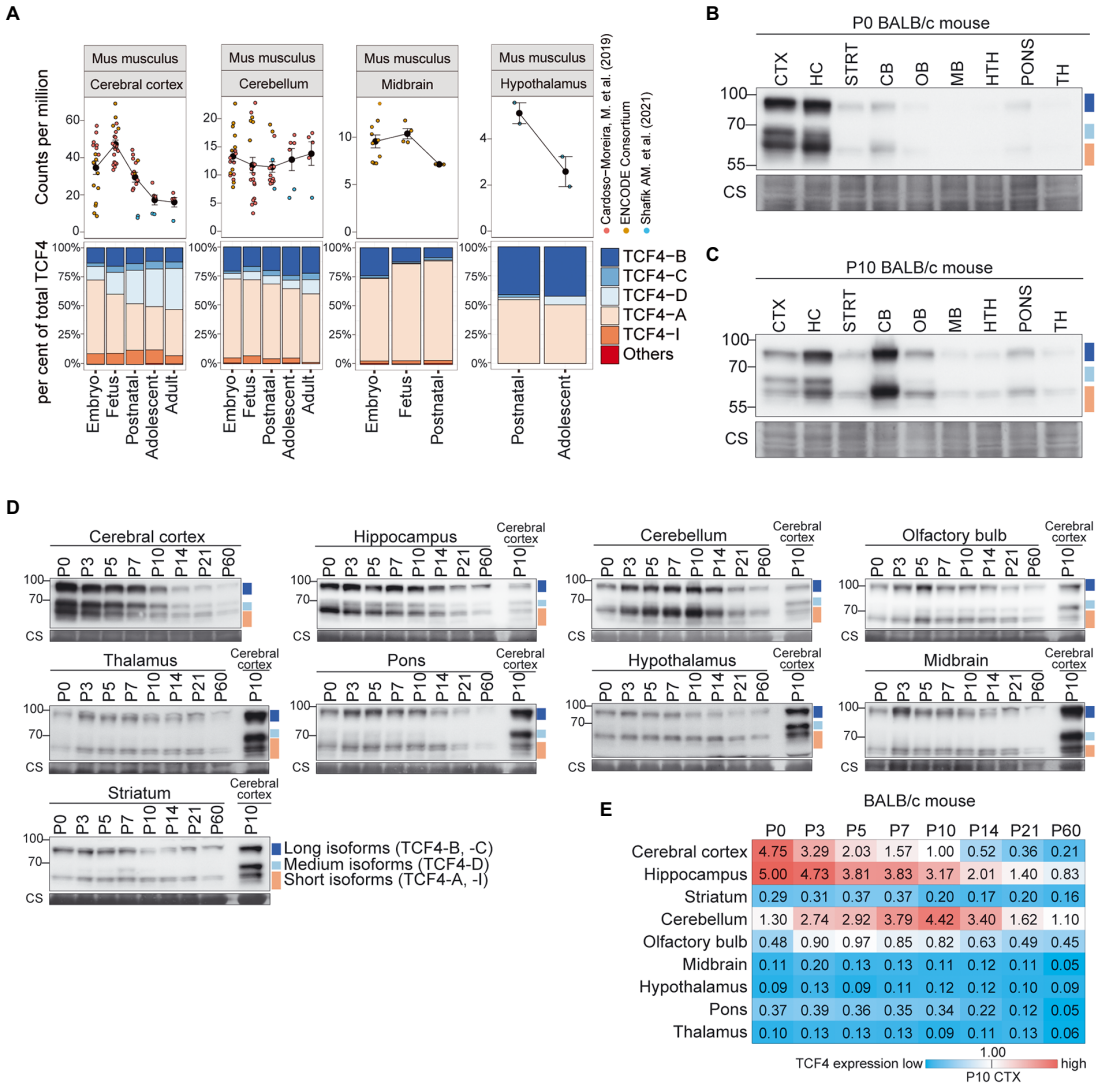
Expression of TCF4 is high in the mouse cerebral cortex, hippocampus and cerebellum. **(A)** Three independent datasets from Cardoso-Moreira et al. (2019), ENCODE Consortium (ENCODE Project Consortium, 2012; Luo et al., 2020), and Shafik et al. (2021) were combined for meta-analysis of *Tcf4* mRNA expression in mouse cerebral cortex, cerebellum, midbrain, and hypothalamus throughout development. mRNA expression of total *Tcf4* is visualized as a line chart (upper panel) where the solid line connects the mean of *Tcf4* expression for each developmental stage and error bars represent standard error of the mean (SEM). The distribution of isoform-specific transcripts is shown as bars (lower panel). Each isoform is represented with different color, as shown in the legend on the right. (**B–D**) Western blot analysis of TCF4 protein expression in different brain areas of BALB/c mouse at P0 **(B)**, P10 **(C)**, and throughout postnatal development **(D)**. The examined brain areas are shown on the top of each panel together with the day of postnatal development. P10 cerebral cortex was used for normalization **(D)**. Coomassie membrane staining (CS) shown at the bottom of each western blot was used as a loading control. The locations of TCF4 isoform groups are color coded and shown on the right. In each panel, molecular weight is shown on the left in kilodaltons. **(E)** TCF4 signals from western blot analysis of different brain areas of BALB/c mouse were quantified and normalized using Coomassie staining. The normalized signal from P10 cerebral cortex was set as 1, and the quantification results are visualized as a heatmap. Color scale gradient represents the relative TCF4 expression level, where blue and red color represents the lowest and the highest total TCF4 protein level, respectively. The studied brain regions are shown on the left and developmental stages on top. CTX, cerebral cortex; HC, hippocampus; CB, cerebellum; STRT, striatum; OB, olfactory bulb; MB, midbrain; HTH, hypothalamus; TH, thalamus; P, postnatal day; and CS, Coomassie staining.

In the mouse brain, *Tcf4* mRNA expression was highest in the cerebral cortex, followed by the cerebellum, midbrain and hypothalamus (Figure 4A; Supplementary Figure S3A). A decrease in *Tcf4* mRNA expression after birth was seen for all the studied brain regions except for the cerebellum, which displayed relatively stable *Tcf4* mRNA levels during postnatal development. The majority of expressed *Tcf4* transcripts encoded isoforms TCF4-B, -C, -D, -A, and -I, with transcripts encoding TCF4-A showing the highest overall expression (Figure 4A; Supplementary Figure S4A) in the mouse brain. The cerebral cortex was the only brain region that displayed a notable change in the expression pattern of transcripts encoding different TCF4 isoforms—during development the expression of TCF4-A decreased and the expression of TCF4-D increased (Figure 4A).

We then sought to describe TCF4 expression at the protein level in mouse brain regions. For this, we dissected the cerebral cortex, hippocampus, cerebellum, striatum, pons, olfactory bulb, hypothalamus, thalamus, and midbrain at eight postnatal stages (P0, 3, 5, 7, 10, 14, 21, and 60) from BALB/C (Figure 4) and C57BL/6 (Supplementary Figure S5) mice, prepared protein lysates and analyzed TCF4 levels by western blot. First, we compared TCF4 protein expression across distinct brain regions at two postnatal stages, P0 and P10 by western blot analysis (Figures 4B,C; Supplementary Figures S5A,B). We observed high TCF4 expression levels in the cerebral cortex and hippocampus at P0, and in the cerebellum at P10 (Figures 4B,C; Supplementary Figures S5A,B). The long and short TCF4 protein isoforms were present in all studied brain regions (Figures 4B,C; Supplementary Figures S5A,B). The medium isoforms had more restricted patterns being detected at high levels in the cerebral cortex and hippocampus, at low levels in the cerebellum, olfactory bulb, and pons, and were below the detection limits in other brain regions (Figures 4B,C).

Next, we focused on the developmental dynamics of TCF4 protein expression in all the dissected mouse brain regions during postnatal development (P0-60; Figure 4D; Supplementary Figure S5C). To better compare TCF4 signals between individual brain regions across development, we used tissue lysate from the P10 cerebral cortex in each experiment as a calibrator and quantified the results (Figure 4E; Supplementary Figure S5F). In agreement with our direct comparisons (Figures 4B,C; Supplementary Figures S5A,B), the highest levels of TCF4 expression were observed in the cerebral cortex, hippocampus, and cerebellum, with all the other studied brain regions showing either moderate (olfactory bulb) or low (striatum, midbrain, hypothalamus, pons, and thalamus) TCF4 expression (Figure 4E; Supplementary Figure S5F). While in the cerebral cortex and hippocampus TCF4 expression peaked just after birth (Figure 4E; Supplementary Figure S5F), cerebellum displayed a slightly delayed increase in TCF4 expression, peaking around a week after birth (Figure 4E; Supplementary Figure S5F). Notably, cerebellum and hippocampus retained a higher expression of TCF4 for a longer period compared to the cerebral cortex (Figure 4E; Supplementary Figure S5F).

**Figure 5:**
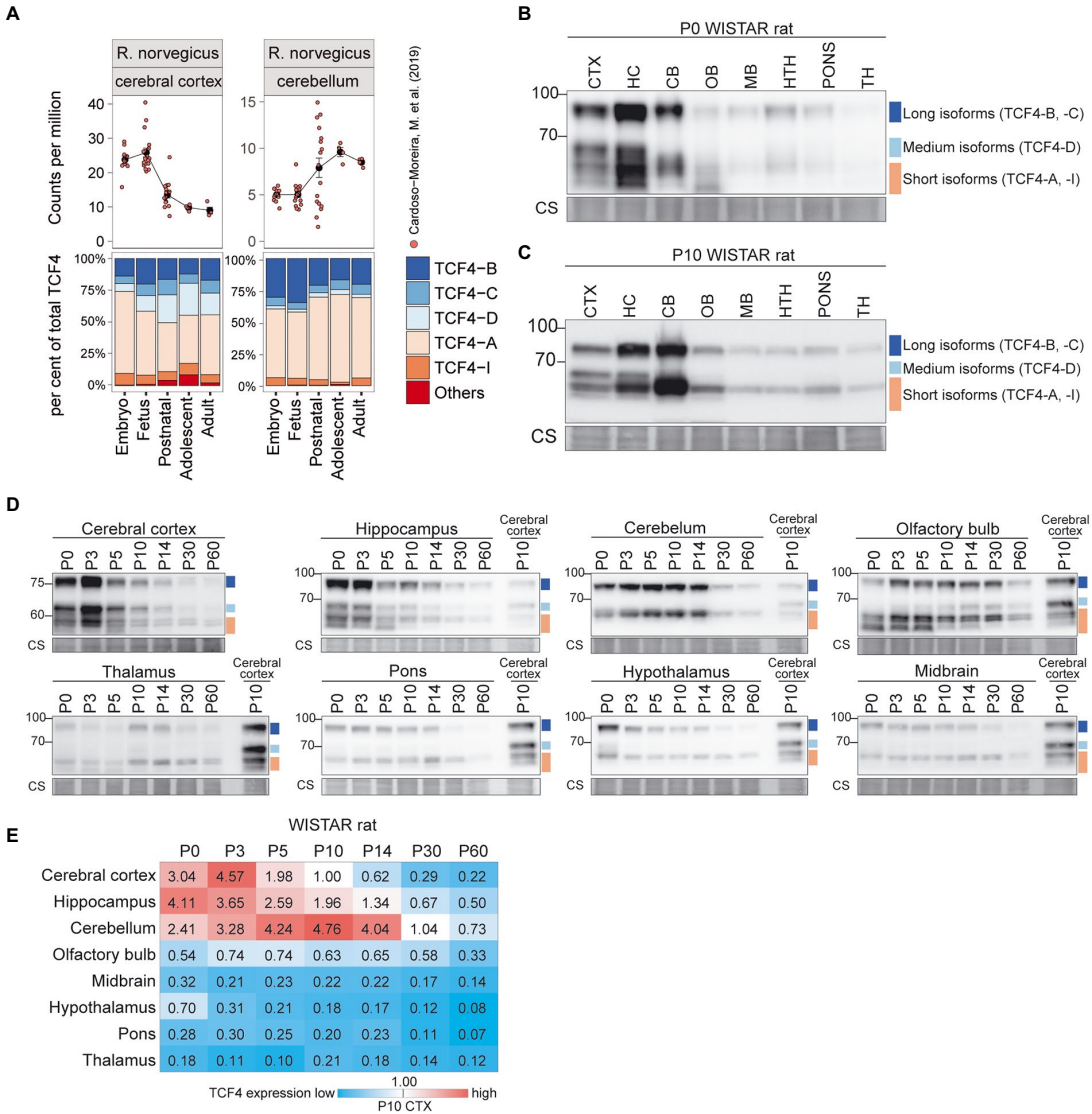
Expression of TCF4 is high in the rat cerebral cortex, hippocampus, and cerebellum. **(A)** Dataset from Cardoso-Moreira et al. (2019) was analyzed for TCF4 expression in rat cerebral cortex and cerebellum throughout development. Total *Tcf4* mRNA expression and the TCF4 isoform distribution was analyzed and visualized similarly to mouse. For more information see legend of Figure 4A. **(B–D)** Western blot analyses of TCF4 protein expression in different brain areas of WISTAR rat at P0 **(B)**, P10 **(C)**, and throughout postnatal development **(D)**. For more details see legend of Figures 4B–D. **(E)** TCF4 signals from western blot analysis of different brain areas of WISTAR rat were quantified, normalized using Coomassie staining, and visualized as a heatmap. For more details see legend of Figure 4E. CTX, cerebral cortex; HC, hippocampus; CB, cerebellum; OB, olfactory bulb; MB, midbrain; HTH, hypothalamus; TH, thalamus; P, postnatal day; and CS, Coomassie staining.

To extend our observations for the mouse, we next characterized TCF4 expression in the brain of another important model organism, the rat (Figure 5). *Tcf4* mRNA expression was highest in the rat cortex, hippocampus, and cerebellum (Figure 5A; Supplementary Figure S3B). At P0 and P10, the highest TCF4 protein expression levels were seen in the cerebral cortex, hippocampus, and cerebellum (Figures 5B,C). The expression dynamics of TCF4 protein isoforms across postnatal development were similar to mouse (Figures 5D,E). Notably, in P0-5 rat cerebral cortex, hippocampus, and olfactory bulb an isoform migrating faster than the TCF4-A isoforms was seen, potentially corresponding to TCF4-I (Figures 5B–D).

Taken together, our data showed that TCF4 expression pattern and dynamics were similar in the mouse and rat brain—the highest TCF4 protein expression was seen in the cerebral cortex and hippocampus around birth, and in the cerebellum 1–2 weeks after birth. TCF4 expression in other brain regions was relatively low. In addition to the differences in overall expression of TCF4, our data revealed that the composition of TCF4 isoforms expressed varies across brain regions in mouse and rat.

### Expression of TCF4 is lower in rodent nonneural organs compared to the brain

**Figure 6:**
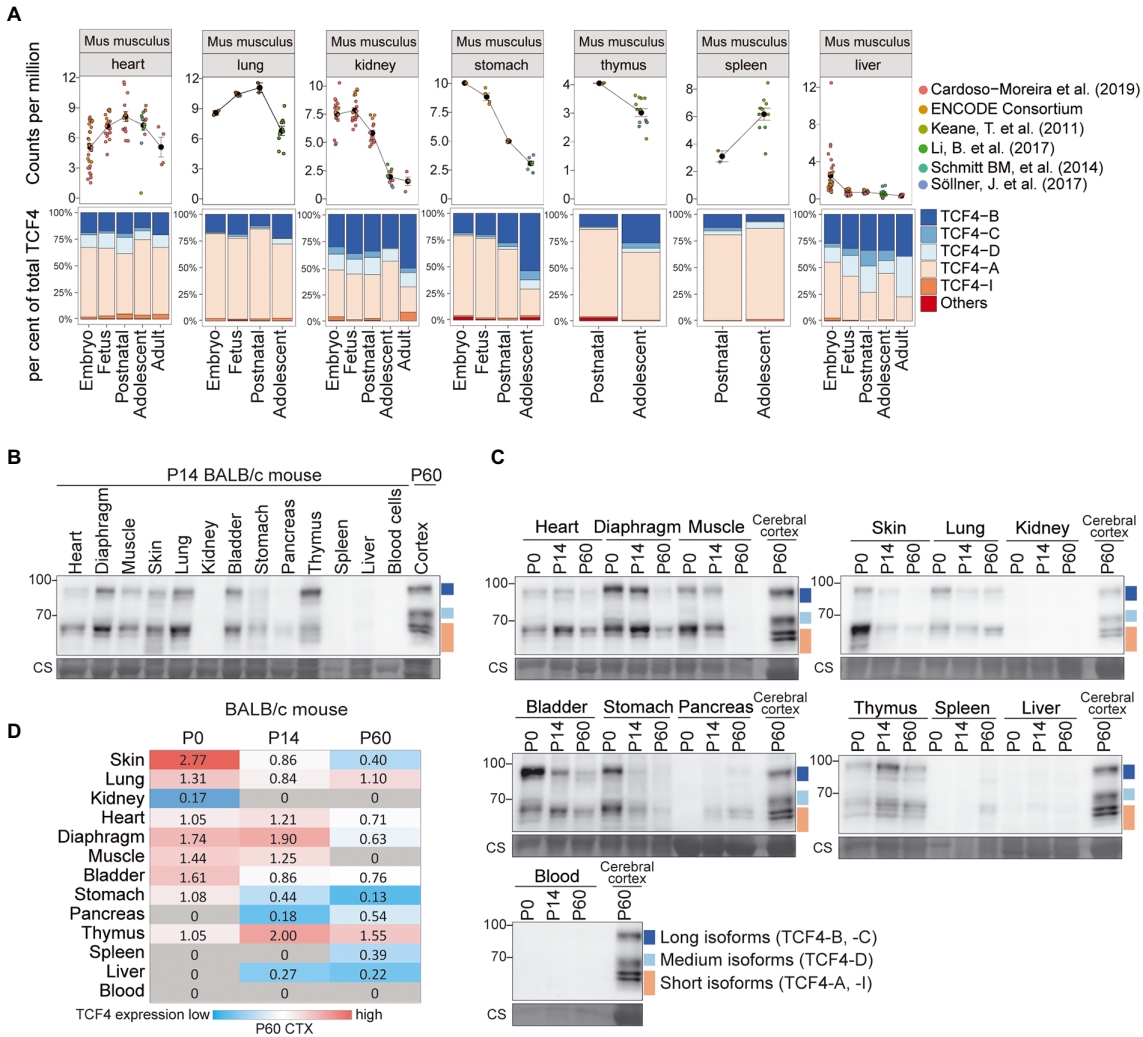
Expression of TCF4 in mouse nonneural tissues. **(A)** Six independent datasets shown on the right were combined for meta-analysis *of Tcf4* expression in mouse heart, lung, kidney, stomach, thymus, spleen, and liver throughout development. Total *Tcf4* levels and the distribution of isoform-specific transcripts is visualized. For more information see legend of Figure 4A. **(B,C)** Western blot analysis of TCF4 protein expression in different nonneural tissues of BALB/c mouse at P14 **(B)**, and throughout postnatal development **(C)**. TCF4 signal from the P60 cerebral cortex was used in each experiment for normalization. Coomassie membrane staining (CS) shown at the bottom of each western blot was used as a loading control. The locations of TCF4 isoform groups are color coded and shown on the right. In each panel, molecular weight is shown on the left in kilodaltons. **(D)** TCF4 signals from western blot analysis of different peripheral tissues of BALB/c mouse were quantified and normalized using Coomassie staining. The signal was then normalized to the signal of the P60 cerebral cortex, and the quantification result is visualized as a heatmap. Color scale gradient represents the relative TCF4 expression level, where blue and red color represents the lowest and the highest total TCF4 protein level, respectively. Gray boxes indicate no detectable TCF4 expression. The studied nonneural tissues are shown on the left and developmental stages on top. P, postnatal day; CS, Coomassie staining.

We then focused on mouse nonneural organs and performed a meta-analysis of available short-read RNA-seq data (Keane et al., 2011; ENCODE Project Consortium, 2012; Schmitt et al., 2014; Yu et al., 2014; Vied et al., 2016; Li et al., 2017; Söllner et al., 2017; Cardoso-Moreira et al., 2019; Luo et al., 2020; Shafik et al., 2021). We analyzed *Tcf4* mRNA expression in the lung, kidney, thymus, spleen, liver, heart, and stomach (Figure 6A; Supplementary Figures S3A, S4A). These tissues displayed comparable *Tcf4* mRNA expression levels except for the liver, where almost no *Tcf4* mRNA expression was seen after birth (Figure 6A; Supplementary Figure S3A). Of the transcripts encoding different TCF4 protein isoforms in nonneural organs, the ones encoding TCF4-A were most prominently expressed, followed by TCF4-B-encoding transcripts (Figure 6A; Supplementary Figure S4A).

Next, we prepared protein lysates from BALB/c (Figure 6) and C57BL/6 (Supplementary Figure S5) mouse heart, diaphragm, muscle, skin, lung, kidney, bladder, stomach, pancreas, thymus, spleen, liver, and blood cells at P0, 14, and 60 for western blot analysis (Figures 6B,C, Supplementary Figure S5). In nonneural tissues, the composition of TCF4 protein isoforms was similar to the one in the brain—both long and short TCF4 protein isoforms were always present, whereas medium-sized TCF4 isoforms were not observed in any of the nonneural tissues (Figures 6B,C; Supplementary Figure S5D,E). Among the studied nonneural tissues, the highest levels of TCF4 protein were seen in the skin at P0 (Figure 6D; Supplementary Figure S5G). Very low TCF4 protein levels were detected in the pancreas, spleen, kidney and liver, and TCF4 protein expression was not seen in the blood cells (Figure 6D; Supplementary Figure S5G).

**Figure 7:**
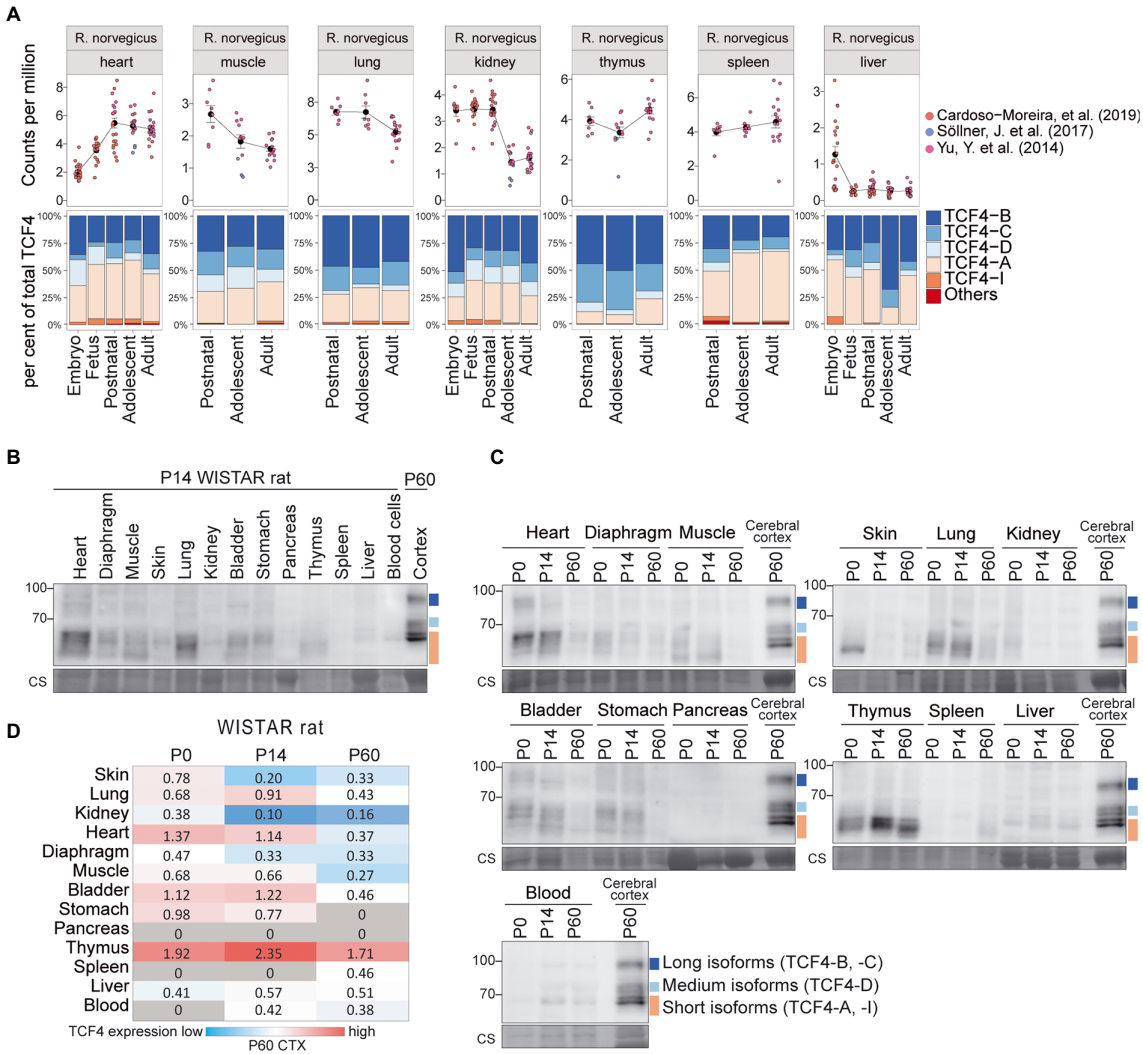
Expression of TCF4 in rat nonneural tissues. **(A)** Three independent datasets shown on the right were combined for meta-analysis of *Tcf4* expression in rat heart, muscle, lung, kidney, thymus, spleen, and liver throughout development. Total *Tcf4* levels and the distribution of isoform-specific transcripts is visualized. For more information see Figure 4A. (**B,C**) Western blot analyses of TCF4 protein expression in different peripheral tissues of WISTAR rat at P14 **(B)**, and throughout postnatal development **(C)**. For more details see legend of Figures 6B,C. **(D)** TCF4 signals from western blot analysis of different nonneural tissues of WISTAR rat were quantified, normalized using Coomassie staining, and visualized as a heatmap. For more details see legend of Figure 6D. P, postnatal day; CTX, cerebral cortex.

We also investigated TCF4 expression in rat nonneural tissues (Figure 7; Supplementary Figures S3B, S4B). Rat *Tcf4* mRNA expression was comparable in all the nonneural tissues except for the liver, where *Tcf4* expression was very low (Figure 7A; Supplementary Figure S3B). Different from mouse, transcripts encoding TCF4-A did not account for the majority of rat *Tcf4* transcripts expressed in nonneural tissues, as also high expression of transcripts encoding TCF4-B and TCF4-C were present (Figure 7A; Supplementary Figure S4B).

Western blot analysis of rat nonneural tissues showed that different from mouse, TCF4 protein expression levels were more uniform between tissues (Figures 7B–D). In rat, TCF4 protein expression was highest in the thymus and was not observed in the pancreas (Figures 7B–D). The expression pattern of TCF4 isoforms in rat nonneural tissues was similar to mouse, i.e., mainly long and short TCF4 isoforms being present (Figures 7B,C).

Overall, the expression of TCF4 in the rodent nonneural tissues was much lower compared to the expression levels observed in the early postnatal development of the central nervous system. In addition, medium-sized TCF4 protein isoforms were almost non-existent in rodent nonneural tissues.

### Expression of TCF4 in human tissues is highest around birth

**Figure 8:**
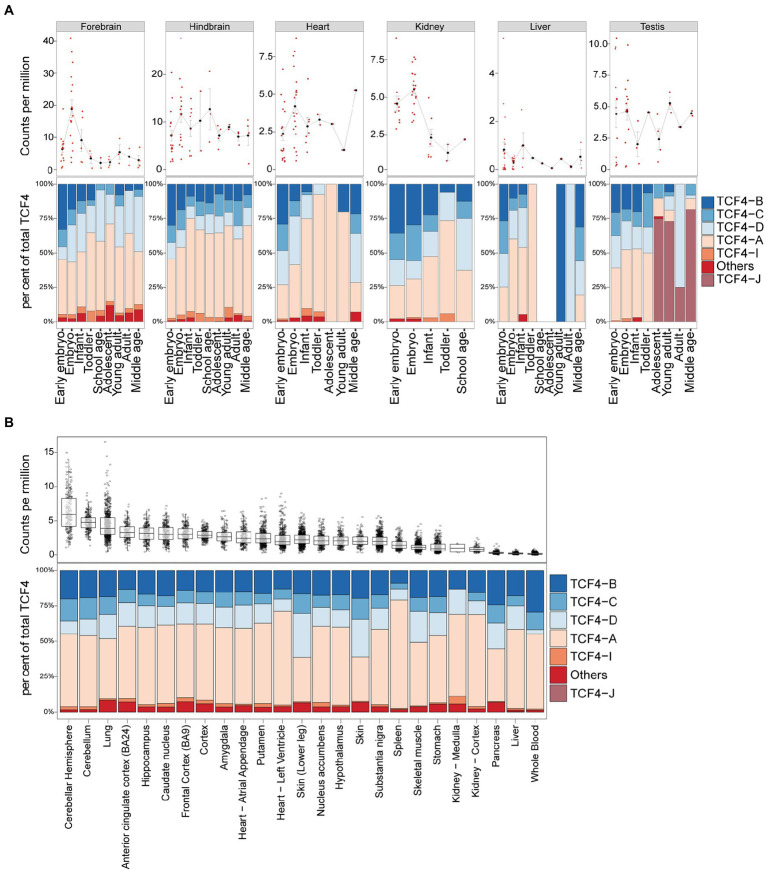
*TCF4* transcripts encoding TCF4-A account for the majority of total *TCF4* expression in the human brain and nonneural tissues. Data from Cardoso-Moreira et al. **(A)** (Cardoso-Moreira et al., 2019) and the Genotype-Tissue Expression (GTEx) project **(B)** was analyzed for *TCF4* expression in the brain and nonneural tissues in humans through development **(A)** or in adults **(B)**. mRNA expression of total *TCF4* is visualized either as a line chart **(A)** or box plot **(B)**, and the distribution of isoform-specific transcripts is shown as bars **(A,B)**. **(A)** Average values are presented as dots and error bars represent SEM. **(B)** The hinges show 25 and 75% quartiles, the horizontal line shows the median value, the upper whisker extends from the hinge to the largest value no further than 1.5 of the inter-quartile range from the hinge, the lower whisker extends from the hinge to the smallest value at most 1.5 * inter-quartile range of the hinge. Each isoform is represented with different color, as shown in the legend on the right. Individual data points are presented as small dots.

Next, we analyzed available short-read RNA-seq data to describe *TCF4* total and isoform-specific mRNA expression in humans. We first analyzed the dataset published by Cardoso-Moreira and colleagues, which contained RNA-seq data from the human brain, heart, kidney, liver, and testis (Cardoso-Moreira et al., 2019). Of the noted tissues, the highest TCF4 mRNA expression was detected in the brain (Figure 8A). Human nonneural tissues showed detectable but lower *TCF4* mRNA levels compared to the brain, especially in the earlier stages of development (Figure 8A). Very low *TCF4* mRNA expression was noted for the liver (Figure 8A). In contrast to other tissues where *TCF4* mRNA levels were relatively stable throughout development, *TCF4* mRNA expression in the forebrain and kidney peaked during prenatal development (Figure 8A). We then used developmental transcriptome data from the BrainSpan project^7^ to describe the changes in total *TCF4* mRNA expression in different brain regions during human development (Supplementary Figure S6). Results were similar in all brain regions—*TCF4* mRNA expression peaked during embryonic development and decreased after birth (Supplementary Figure S6).

Next, we conducted a similar analysis for adult human RNA-seq data from the Genotype-Tissue Expression (GTEx) project.^8^ A selection of adult human tissues is shown in Figure 8B and all the studied tissues can be found in Supplementary Figure S7. When comparing different adult human tissues, the highest *TCF4* mRNA expression levels were seen in the adult human brain and adipose tissues (Figure 8B; Supplementary Figure S7). Almost no *TCF4* mRNA was detected in the human pancreas, liver, and whole blood (Figure 8B; Supplementary Figure S7).

For both Cardoso-Moreira et al. and GTEx datasets, transcripts encoding TCF4-A made up around 50% of the total TCF4 mRNA levels in all the studied tissues, with the only exception being the testis (Figures 8A,B; Supplementary Figure S7). In the human testis, mRNA transcripts encoding TCF4-J accounted for the majority of total *TCF4* levels beginning from adolescence, which coincides with the start of spermatogenesis (Figure 8A; Supplementary Figure S6). The other major isoform-specific transcripts expressed in human tissues were TCF4-B, -C, and-D (Figures 8A,B; Supplementary Figure S6).

**Figure 9:**
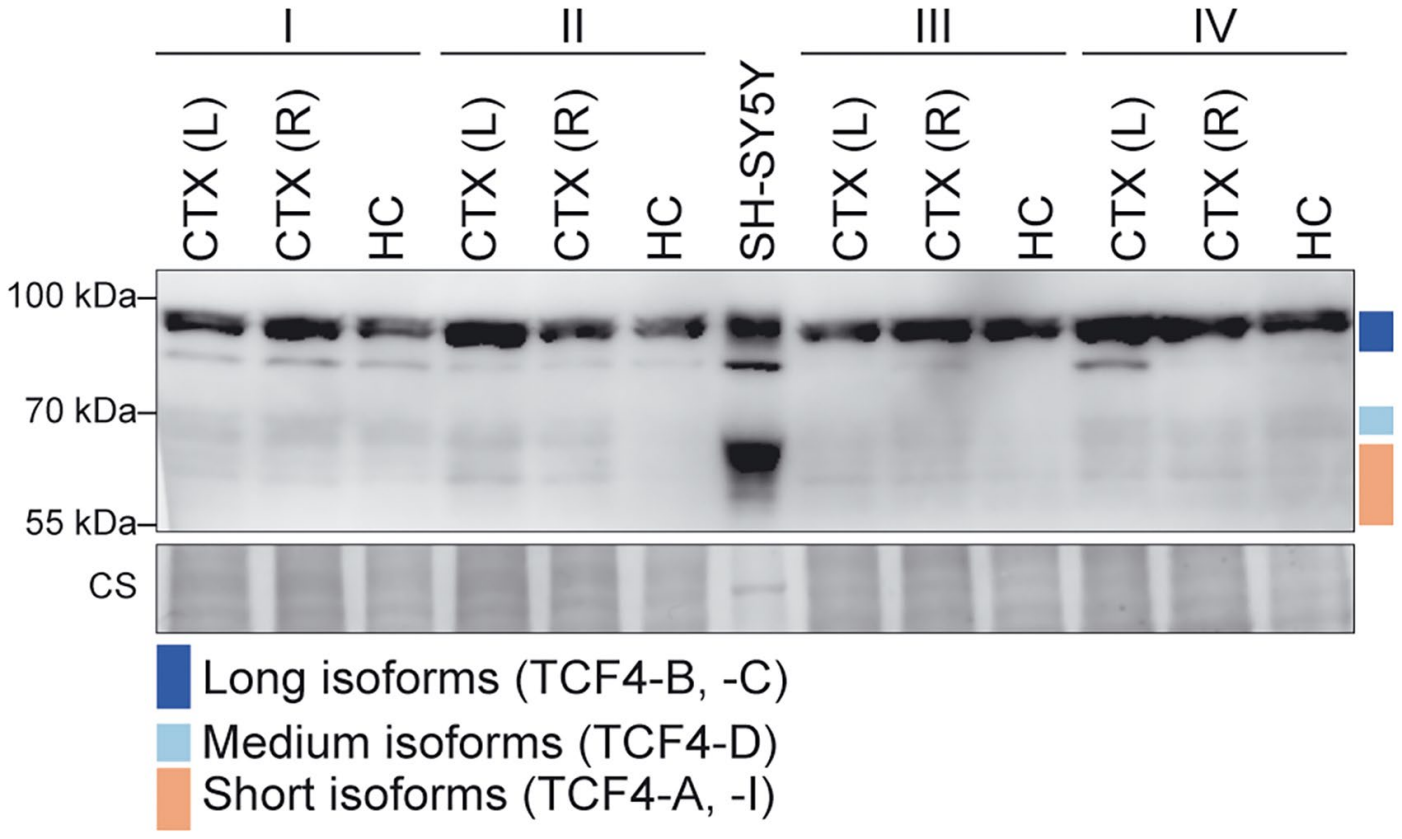
Expression of TCF4 protein isoforms in adult human cerebral cortex and hippocampus. Western blot analysis of TCF4 protein expression in the adult human cerebral cortex left (L) and right (R) hemisphere, hippocampus, and SH-SY5Y cell line. Samples from four individuals aged 62 (I), 65 (II), 67 (III), and 70 (IV) yearswere used. Coomassie staining (CS) shown at the bottom was used as loading control. The locations of TCF4 isoform groups are color coded and shown on the right. In each panel, molecular weight is shown on the left in kilodaltons. CS, coomassie staining.

Next, we aimed to investigate TCF4 isoform composition in the adult human cerebral cortex and hippocampus. For this, we prepared protein lysates from these brain regions and human neuroblastoma cell line SH-SY5Y used for isoforms’ mobility comparison. Western blot analysis revealed that TCF4 protein signal was detectable in both adult human brain and SH-SY5Y cell line (Figure 9). We also detected a possible non-specific signal located between the long and medium TCF4 isoforms in both SH-SY5Y and human brain lysates (Figure 9) since this signal was not detected using other TCF4 antibodies (data not shown) validated by us before (Nurm et al., 2021). Different to SH-SY5Y cell line, we detected all three TCF4 isoform groups in the adult human brain, however expression level of longer TCF4 isoforms was higher compared to the medium and short isoforms (Figure 9; Supplementary Figure S8). This result was on the contrary with protein isoform patterns seen in the rodent brain and the results from our human RNA-seq data analysis, which could result from protein stability, post-mortem artifacts or signal masking by other similar-sized proteins. Nevertheless, the presence of long, medium and short TCF4 isoforms in adult human brain matched with TCF4 isoform pattern in rodents, however the species-and tissue-specific temporal expression dynamics of different TCF4 isoforms during the development cannot be emphasized more.

Altogether, our results show that *TCF4* mRNA is expressed at high levels in the human brain during development and the expression is retained in the adulthood. In most tissues transcripts encoding for TCF4-A were the most prominent ones, while in the testis TCF4-J encoding transcripts were mostly expressed. In adult human brain, long, medium and short TCF4 protein isoforms are expressed.

## Discussion

Transcription factor TCF4 has been extensively studied due to its linkage with neurocognitive disorders such as intellectual disability, schizophrenia and Pitt-Hopkins syndrome (Stefansson et al., 2009; Kharbanda et al., 2016; Zollino et al., 2019). Knowledge of TCF4 expression across tissues and development would lay the foundation to understanding how these diseases develop and may help with the generation of gene therapy applications for the many TCF4 associated diseases. Transcripts from the mouse and human *TCF4* gene have been previously described in our lab using mRNA and expressed sequence tag data from public databases (Sepp et al., 2011; Nurm et al., 2021). Short read RNA-seq data can also be used to describe *Tcf4* transcripts. However, due to the structure of the *Tcf4* gene, it can be complicated to describe expression of transcripts encoding different isoforms based only on short read RNA-seq data as splicing features or 5′ exons can be difficult to detect. Our direct long-read RNA-seq analysis of *Tcf4* transcripts in the rodent brain revealed that transcription from the *Tcf4* gene results in transcripts encoding 5 N-terminally distinct TCF4 protein isoforms in the rodent brain – TCF4-B, -C, -D, -A, and-I. This result falls in line with previous observations by Nurm et al. (2021).

Expression of total *Tcf4* mRNA during development has been extensively studied mainly in the mouse cerebral cortex at the total mRNA level, with the highest expression reported around birth (E16-P6; Li et al., 2019; Phan et al., 2020). This is in accordance with our RNA-seq meta-analysis and applies for both mouse and rat. In addition, we show that the expression dynamics of *Tcf4* in the rodent brain and nonneural tissues are similar—highest *Tcf4* expression can be detected around birth, followed by a decline during postnatal development. However, studying only total *Tcf4* mRNA levels provides only partial information about *Tcf4* expression as transcription from the *Tcf4* gene results in numerous transcripts, and encoded protein isoforms have different functional protein domains and transactivation capability (Sepp et al., 2011, 2017; Nurm et al., 2021). We have previously developed a method which quantifies the expression of different *Tcf4* protein isoform-encoding transcripts using short read RNA-seq data (Sirp et al., 2020). Here, we applied the same approach to describe the expression of different TCF4 isoforms throughout development using previously published RNA-seq data. When leaving aside the great increase in the expression of TCF4-J in the adolescent human testis (transcripts encoding TCF4-J are not present in rodents), no drastic change concerning switching from the expression of one TCF4 isoform to the other was detected during the rodent and human development. This suggests that the same TCF4 isoforms that are necessary during development may also be vital for the TCF4-mediated normal functioning of the adult organism.

The necessity of so many different TCF4 isoforms remains unknown. In humans, mutations in the 5′ region of *TCF4* gene, which affect only the longer isoforms, lead to mild–moderate intellectual disability (Kharbanda et al., 2016). As the resulting disease is not as severe as the Pitt-Hopkins syndrome, it may mean that a slight decrease in overall TCF4 expression causes the phenotype. However, it is also possible that the longer TCF4 isoforms have specific functions which cannot be compensated by other TCF4 isoforms, and mutations affecting only a subset of TCF4 isoforms result in less severe effects than seen for mutations affecting all the isoforms. Recently, it has been shown that postnatal normalization of TCF4 expression to wild type levels can rescue the phenotype of TCF4 heterozygous knockout mice (Kim et al., 2022). In addition, studies of Daughterless, the orthologue of TCF4 in the fruit fly, have shown that it is possible to partially rescue the severe embryonic neuronal phenotype of Daughterless null mutation by overexpressing either human TCF4-A or TCF4-B (Tamberg et al., 2015). The generation of TCF4 isoform-specific mutant mice would help to identify whether TCF4 isoforms have distinct or similar functions. Such a model could be used to determine whether it is possible to rescue the negative phenotype resulting from a knock-out of a single TCF4 isoform by increasing the level of an another TCF4 isoform. However, generating such a model comes with many challenges. To begin with, it can be complicated to silence all the TCF4 isoforms individually by causing just frameshift mutations as only some isoforms (e.g., TCF4-B and-A) have their translation start sites located in independent 5′ exons. In addition, mutating one *TCF4* transcript can result in the upregulation of another *TCF4* transcript – an effect that we saw when silencing TCF4-A in Neuro2a cells that resulted in an increase in TCF4-I levels. We have also previously shown that Fuchs’ Endothelial Dystrophy-related endogenous downregulation of transcripts encoding longer TCF4 isoforms results in the upregulation of shorter isoforms (Sirp et al., 2020).

To fully characterize TCF4 expression, it is important to consider all TCF4 protein isoforms. Previously, a large study on the expression of TCF4 protein during neurodevelopment has been performed by Matthias Jung and colleagues using immunohistochemical analysis with an antibody specific only for the long TCF4 protein isoforms (Jung et al., 2018). Another study by Kim and colleagues used TCF4-GFP reporter mice to characterize total TCF4 expression in the mouse brain (Kim et al., 2020). A major limitation of these methods is that they cannot be used to describe the expression of different TCF4 protein isoforms. Overall, our results of total TCF4 protein expression levels during postnatal development of different mouse brain areas agree with the previously reported data. However, by using a TCF4 antibody specific for all the TCF4 isoforms in western blot analysis, we were able to distinguish TCF4 protein expression in three different groups – long (TCF4-B, TCF4-C), medium (TCF4-D) and short TCF4 isoforms (TCF4-A, TCF4-I). Isoform-specific silencing of TCF4 and *in vitro* translated TCF4 protein isoforms confirmed the locations of TCF4 isoforms in western blot. However, it should be noted that a similar pattern of TCF4 isoforms in western blot analysis between different tissues may not necessarily indicate the presence of exactly the same TCF4 isoforms. We acknowledge that *in vitro* and *in vivo* translated proteins can migrate differently in western blot analysis due to the differences in post-translational modifications of the proteins in various cell types.

The expression dynamics of TCF4 during the development varied in different brain regions. In contrast to the cerebral cortex where TCF4 expression levels decline after birth, in the cerebellum, hippocampus and olfactory bulb we saw a more prolonged high TCF4 protein expression. In the cerebellum TCF4 protein expression peaks about a week later than in any of the other brain regions. While the majority of the neurogenesis in the central nervous system happens during prenatal development, the granule cell precursors of the cerebellum and olfactory bulb, and the dentate gyrus of the hippocampus proliferate and differentiate after birth (Chen et al., 2017), where TCF4 was shown to be highly expressed (Jung et al., 2018; Kim et al., 2020), and regulate the maturation of the cerebellar granule cells (Kim et al., 2020). We propose that high TCF4 expression is necessary for the maturation of distinct brain regions, whereas fully developed brain areas display low and stable TCF4 expression necessary for normal function of the adult nervous system.

Expression of long and short TCF4 protein isoforms was seen in all brain regions and nonneural tissues where TCF4 was detectable. However, in rodents the medium TCF4 isoforms (TCF4-D) were only observed in the brain, specifically in the cerebral cortex, hippocampus, and olfactory bulb. Interestingly, in the whole rodent brain the expression of medium isoforms became apparent only in later stages of embryonic development. The only well-known functional protein domain located in the N-terminal region of longer TCF4 isoforms is activation domain 1. While the long TCF4 isoforms (TCF4-B and-C) contain this domain, TCF4-D lacks it. In addition, different from short TCF4 isoforms (TCF4-A and-I), TCF4-D contains a nuclear localisation signal. It remains to be studied what the function of TCF4-D in the development of the nervous system is and why this TCF4 isoform is missing in the cerebellum where TCF4 is otherwise highly expressed.

Based on the results of the present study we propose that a mixture consisting of TCF4-B, -C, -D, -A, and-I encoding constructs could be used in gene therapy approaches for Pitt-Hopkins syndrome. However, it should be noted that TCF4 expression levels vary between brain regions and cell types during development (Jung et al., 2018; Kim et al., 2020), suggesting that the dosage of TCF4 isoforms needs to be highly regulated. The direct administration of a cocktail of TCF4 isoforms may allow easier control of each isoform compared to other gene therapy approaches such as activation of endogenous promoters and enhancers. As a next step of this study, a similar TCF4 protein expression analysis should be done for human brain regions with a focus on the hippocampus and cerebral cortex, as studies of structural brain anomalies in PTHS-patients and *Tcf4*-heterozygous mice have shown hypoplasia of these brain regions (Marangi and Zollino, 2015). In addition, the expression of TCF4 different transcripts and the protein isoforms they encode should be studied at the single cell level to better understand how the many TCF4 isoforms are regulated between cell types.

## Data availability statement

The datasets analyzed and presented in this study can be found in online repositories and in the Supplementary material. The names of the repository/repositories and accession number(s) can be found in the article.

## Ethics statement

The studies involving human participants were reviewed and approved by Tallinn Committee for Medical Studies, National Institute for Health Development (Permit Number 402). The patients/participants provided their written informed consent to participate in this study. The animal study was reviewed and approved by Ministry of Agriculture of Estonia (Permit Number: 45).

## Author contributions

ASi and JT designed research, performed research, analyzed data, and wrote the paper. ASh, LT, and CK performed research, analyzed data, and wrote the paper. LK performed research. TT designed research, wrote the paper, and acquired funding. All authors contributed to the article and approved the submitted version.

## Funding

This study was supported by Estonian Research Council (grant PRG805), European Union through the European Regional Development Fund (project no. 2014-2020.4.01.15-0012) and H2020-MSCA-RISE-2016 (EU734791), and Pitt Hopkins Research Foundation (grant no. 21). The funding sources were not involved in study design, analysis and interpretation of data, writing of the report and in the decision to submit the article for publication.

## Conflict of interest

JT and TT were employed by Protobios LLC.

The remaining authors declare that the research was conducted in the absence of any commercial or financial relationships that could be construed as a potential conflict of interest.

## Publisher’s note

All claims expressed in this article are solely those of the authors and do not necessarily represent those of their affiliated organizations, or those of the publisher, the editors and the reviewers. Any product that may be evaluated in this article, or claim that may be made by its manufacturer, is not guaranteed or endorsed by the publisher.

## Footnotes

^1^ https://genome.ucsc.edu

^2^ https://benchling.com

^3^ http://www.addgene.org/crispr/zhang/

^4^ https://github.com/CSKiir/Sirp_et_al_2022

^5^ https://gtexportal.org

^6^ http://brainspan.org

^7^ http://brainspan.org

^8^ https://gtexportal.org

## Abbreviations

TCF4: Transcription factor 4
RNA-seq: RNA sequencing
RT: Room temperature
RT-qPCR: Reverse transcription quantitative PCR
gRNA: Guide RNA
P: Postnatal
E: Embryonic
CTX: Cerebral cortex
HC: Hippocampi
CB: Cerebellum
OB: Olfactory bulb
HTH: Hypothalamus
MB: Midbrain
TH: Thalamus
STRT: Striatum
