# Supplementary material for: Expression of alternative transcription factor 4 mRNAs and protein isoforms in the developing and adult rodent and human tissues
Source: Front Mol Neurosci. 2022 Nov 2;15:1033224. doi: 10.3389/fnmol.2022.1033224 (PMC9666405; doi:10.3389/fnmol.2022.1033224)
Supplement: Supplementary file 3 [file Data_Sheet_3.docx]

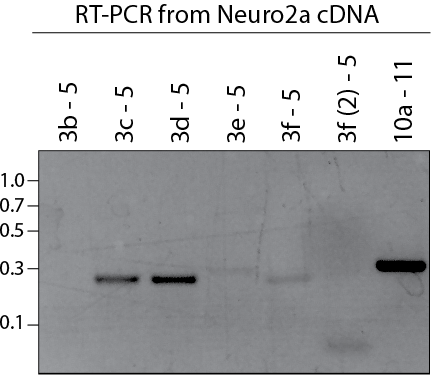


**Supplementary Figure S1. Expression of alternative 5’ exons used in transcripts encoding TCF4-A and TCF4-B in Neuro-2a cells.** RT-PCR analysis of different *Tcf4* 5´exon-containing transcripts from the cDNA of mouse Neuro2a cells. Primer pairs used for the RT-PCR are marked on top of each lane and can be found in supplementary table S1. DNA marker is shown on the left in kbp-s.


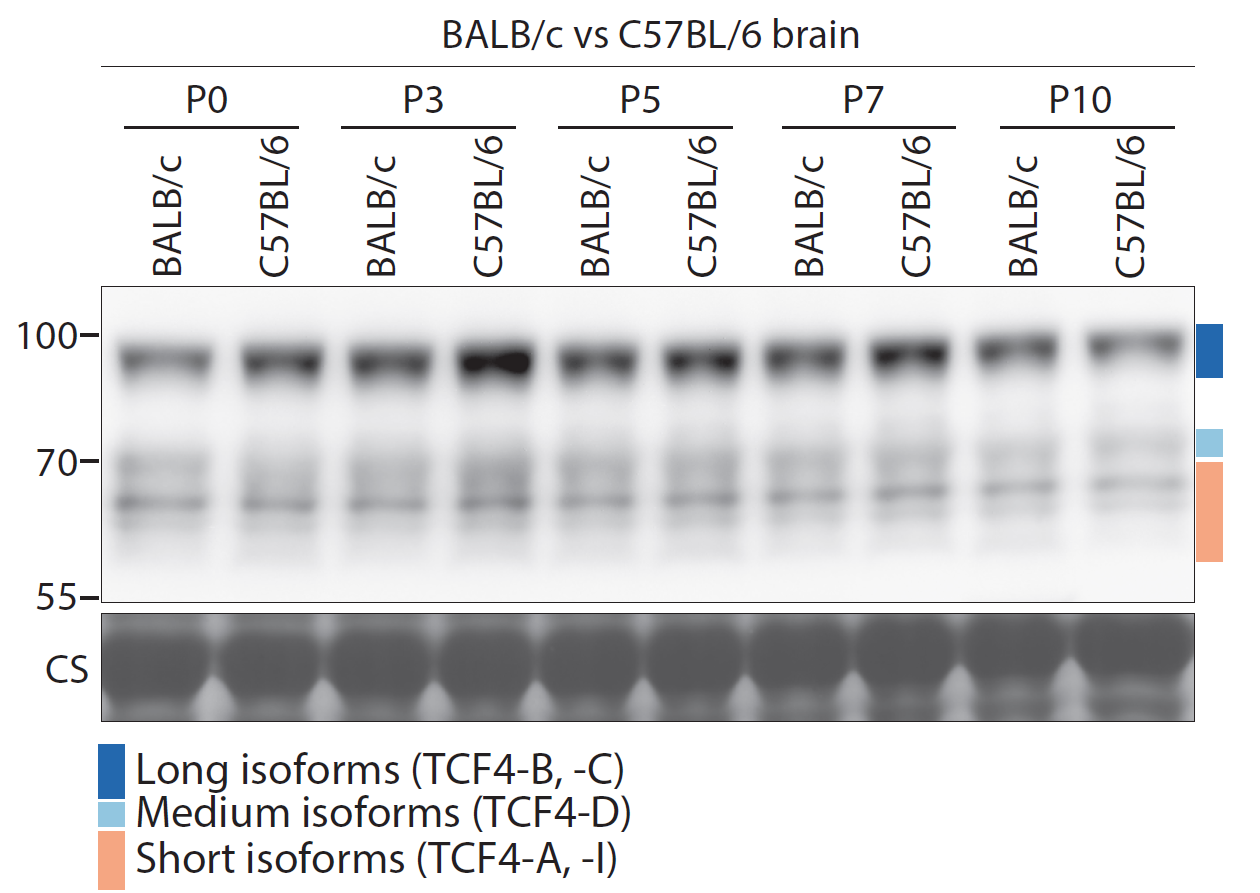


**Supplementary Figure S2. Expression of TCF4 protein in the mouse brain.** Comparison of TCF4 expression in the whole brain of BALB/c and C57BL/6 mouse at P0, 3, 5, 7, and 10 developmental stages. Developmental stage and name of mouse strain is indicated on the top of each lane. Coomassie membrane staining (CS) shown at the bottom was used as loading control. Molecular weight markers are indicated on the left in kilodaltons. TCF4 isoforms were grouped into three – long, medium and short isoforms. The locations of TCF4 isoform groups are colour coded and shown on the right. P, postnatal day; CS, coomassie staining.


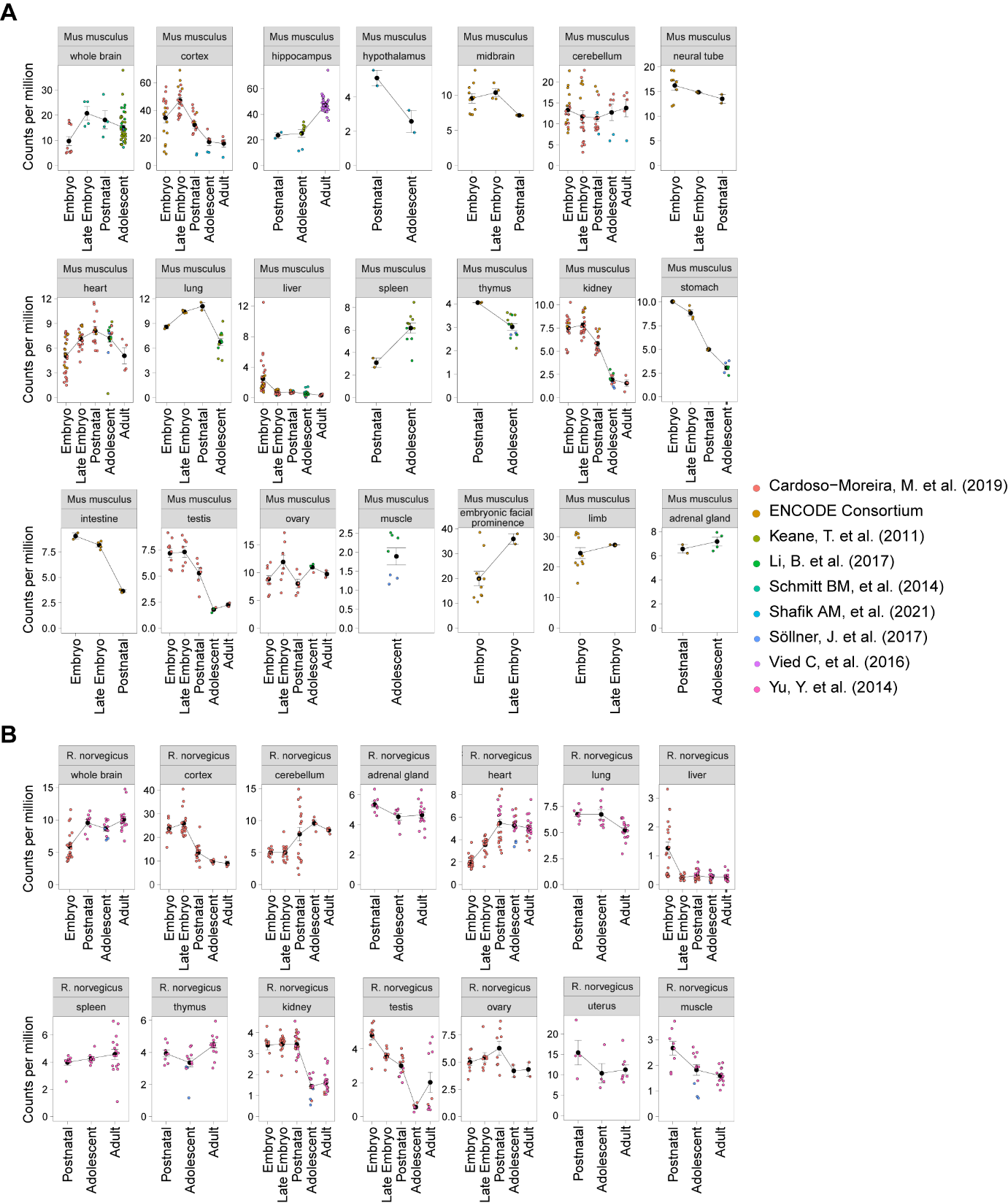


**Supplementary Figure S3. Expression of total *Tcf4* mRNA in different brain regions and nonneural tissues during rodent development.** Nine independent datasets shown on the right were combined for meta-analysis of *Tcf4* expression in the mouse (A) and rat (B) brain and nonneural tissues. mRNA expression of total *Tcf4* is visualised as a line chart where the solid line connects the mean of *Tcf4* expression for each developmental stage and error bars represent SEM.


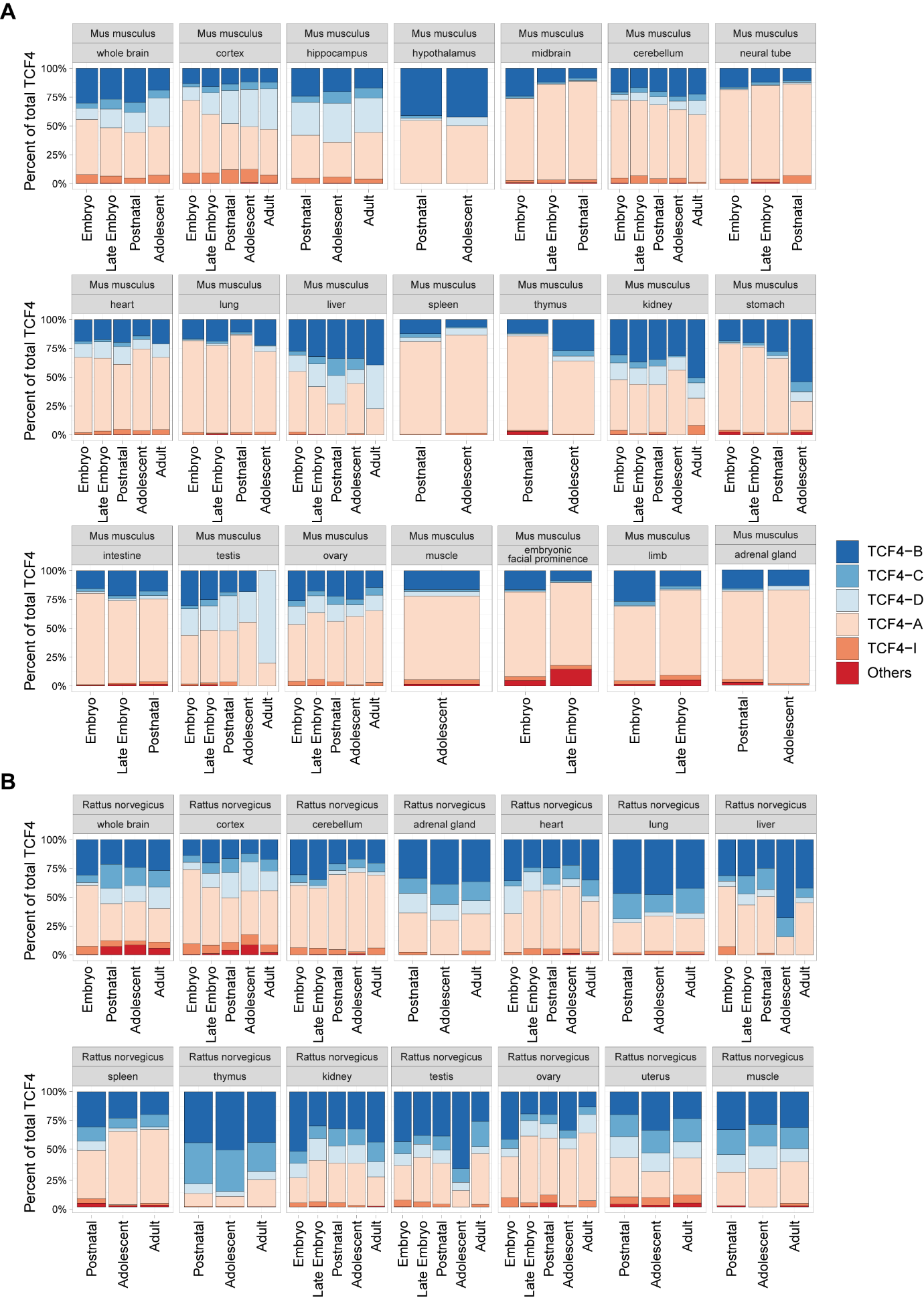
**Supplementary Figure S4. Expression of *Tcf4* isoform-specific transcripts in different brain regions and peripheral tissues during rodent development.** Nine independent datasets shown in Supplementary figure S3 were analysed for the distribution of isoform-specific *Tcf4* transcripts in the mouse (A) and rat (B) brain and nonneural tissues and shown as bars. Each isoform is represented with different colour, as shown in the legend on the right.

**
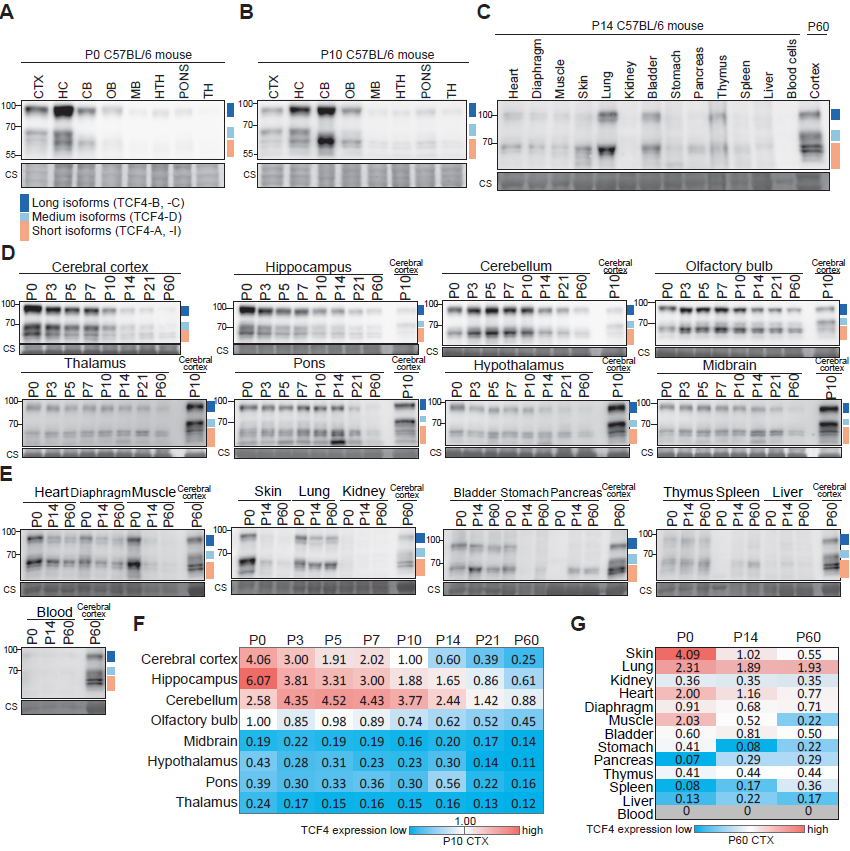
**

**Supplementary Figure S5. Expression of TCF4 protein in C57BL/6 mouse. (A, B, C)** Western blot analysis of TCF4 protein expression in different brain areas of C57BL/c mouse at P0 (A), P10 (B), and in different peripheral tissues at P14 (C). (**D, E**) Western blot analysis of TCF4 protein expression during C57BL/c mouse postnatal development in distinct brain regions (D) and peripheral tissues (E). The examined tissues are shown on the top of each blot together with the day of postnatal development. P10 (brain regions) or P60 (peripheral tissues) cerebral cortex was used for normalization. Coomassie membrane staining (CS) shown at the bottom of each western blot was used as a loading control. The locations of TCF4 isoform groups are colour coded and shown on the right. In each panel, molecular weight is shown on the left in kilodaltons. (**F, G**) TCF4 signals from western blot analysis of different brain areas and peripheral tissues of BALB/c mouse were quantified and normalized using Coomassie staining. The signal was then normalized to the signal of the P10 (brain regions) or P60 (peripheral tissues) cerebral cortex, and the quantification result is visualized as separate heatmaps for the brain (F) and nonneural tissues (G). For heatmap of TCF4 expression in brain regions, the TCF4 signal from P10 cerebral cortex was set as 1. Colour scale gradient represents the TCF4 expression level, where blue and red colour represents the lowest and the highest total TCF4 protein level, respectively. The name of studied tissue is shown on the left and developmental stages on the top of heatmap. CTX, cerebral cortex; HC, hippocampus; CB, cerebellum; STRT, striatum; OB, olfactory bulb; MB, midbrain; HTH, hypothalamus; TH, thalamus; P, postnatal day; CS, Coomassie staining


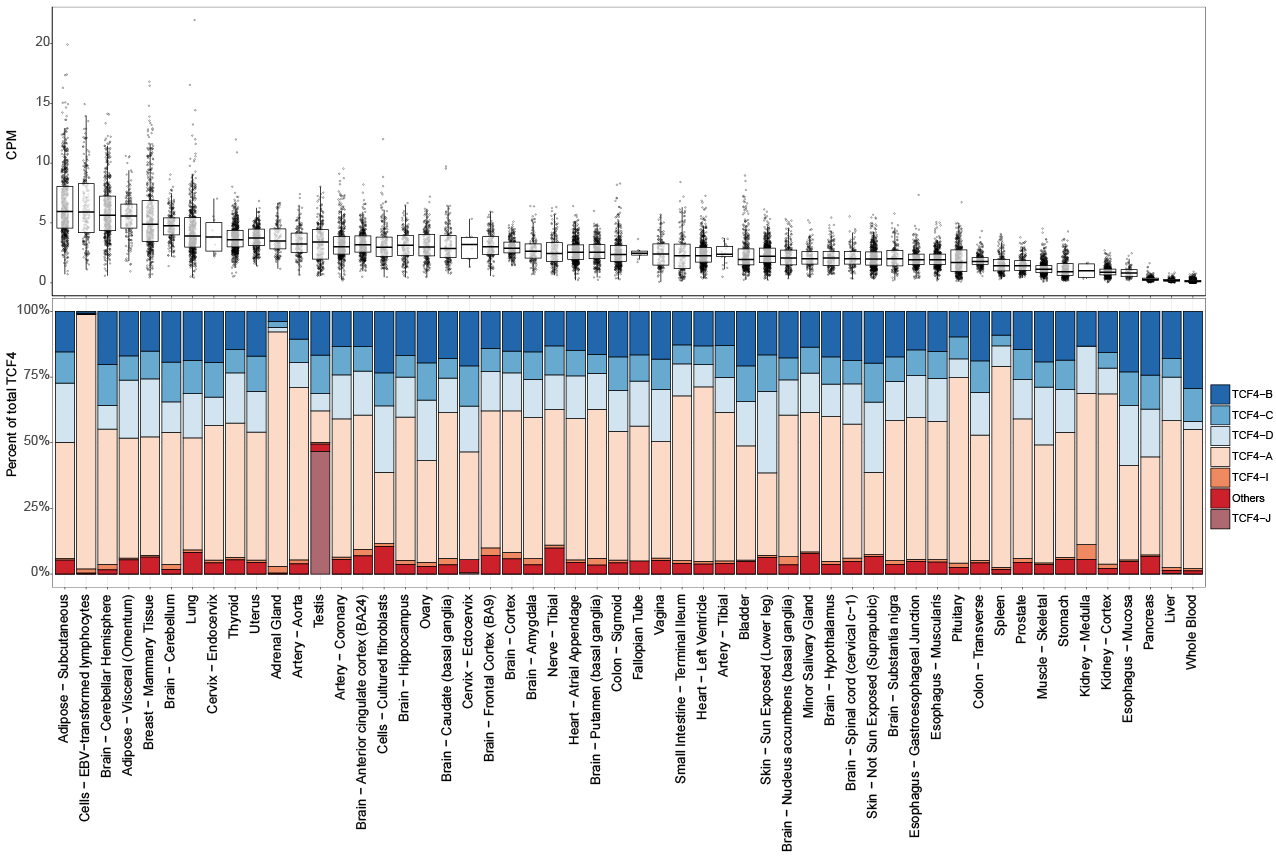
 **Supplementary Figure S6. Expression of total *TCF4* mRNA and *TCF4* isoform-specific transcripts in the adult human brain and nonneural tissues.** Adult human data from the Genotype-Tissue Expression (GTEx) project was analysed for total *TCF4* mRNA expression (visualised as a box plot, upper panel) and for the distribution of isoform-specific transcripts (visualised as bars, lower panel). The hinges show 25% and 75% quartiles, the horizontal line shows the median value, the upper whisker extends from the hinge to the largest value no further than 1.5 * inter-quartile range from the hinge, the lower whisker extends from the hinge to the smallest value at most 1.5 * inter-quartile range of the hinge. Each isoform is represented with different colour, as shown in the legend on the right. Individual data points are presented as small dots.


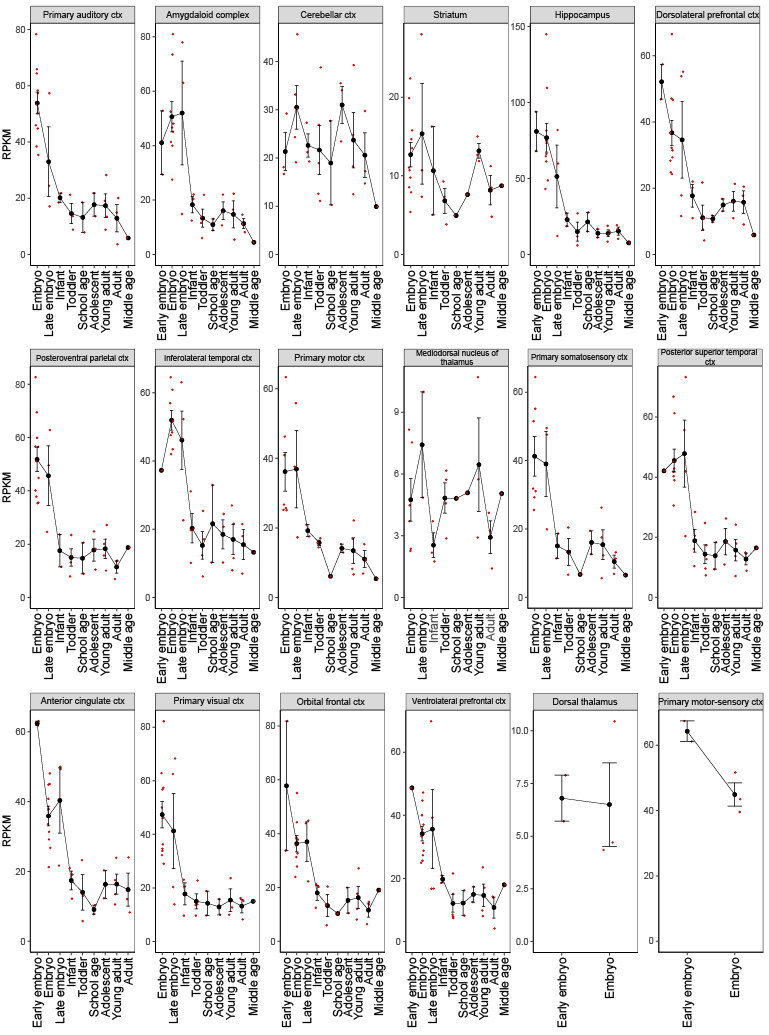


**Supplementary Figure S7. Expression of total *TCF4* mRNA in different brain regions.** Data from the BrainSpan developmental transcriptome RNA-seq was analyzed to describe *TCF4* expression in different brain regions during human development. mRNA expression of total *TCF4* is visualised as a line chart where the solid line connects the mean of *TCF4* expression for each developmental stage and error bars represent SEM. Data is shown as reads per kilobase per million (RPKM), errors bars represent SEM and individual data points are displayed as red dots. CTX – cortex


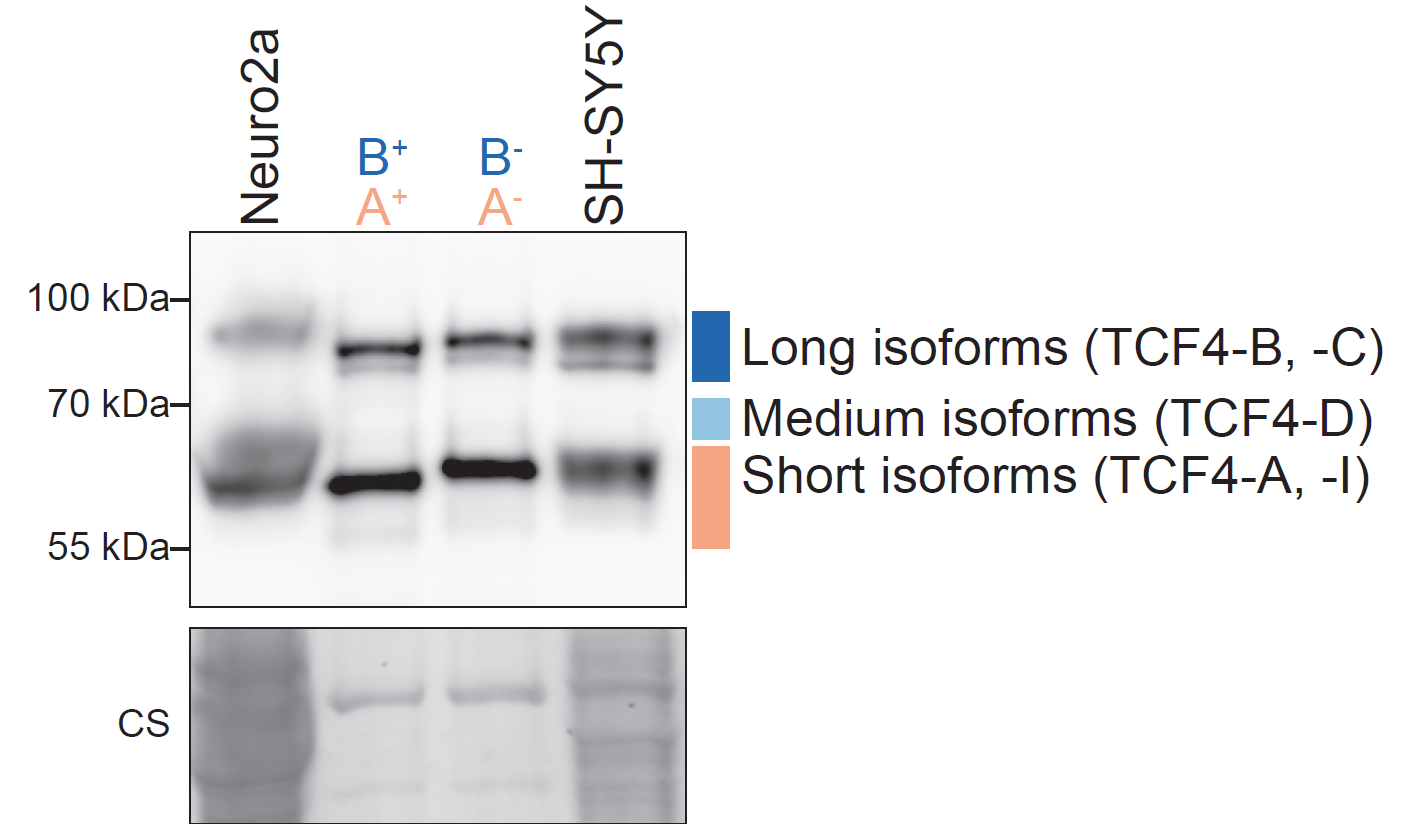


**Supplementary Figure S8. Expression of TCF4 protein isoforms in mouse and human neuroblastoma cell lines.** Comparison of TCF4 protein isoform mobility in the mouse Neuro2a and human SH-SY5Y neuroblastoma cell lines with combinations of *in vitro* translated TCF4 isoforms (shown on the top) in SDS-PAGE. Coomassie membrane staining (CS) shown at the bottom was used as loading control. Molecular weight markers are indicated on the left in kilodaltons. TCF4 isoforms were grouped into three – long, medium and short isoforms. The locations of TCF4 isoform groups are colour coded and shown on the right. CS, coomassie staining.
